# Exercise-stimulated primary cilia on preosteoclasts promote periosteal-bone formation

**DOI:** 10.1038/s12276-026-01765-5

**Published:** 2026-07-01

**Authors:** Jin-Man Kim, Young-Sun Lee, Min Ji Kim, Yewon Kim, Ho Kyoung Kim, Sung-Ah Moon, Hanjun Kim, Han Jin Cho, Sunghoon Kim, Seung Hun Lee, Min-Seon Kim, Young-Bum Kim, Jung-Min Koh

**Affiliations:** 1 Asan Institute for Life Sciences, Asan Medical Center, Seoul, Republic of Korea; 2 AMIST, Asan Medical Center, University of Ulsan College of Medicine, Seoul, Republic of Korea; 3 R&D Center, New Drug Research Institute, Hanlim Pharm. Co., Ltd, Seoul, Republic of Korea; 4 Division of Endocrinology and Metabolism, Asan Medical Center, University of Ulsan College of Medicine, Seoul, Republic of Korea; 5 Division of Endocrinology, Diabetes, and Metabolism, Beth Israel Deaconess Medical Center and Harvard Medical School, Boston, MA USA

**Keywords:** Mechanisms of disease, Bone development, Central control of bone remodelling

## Abstract

Exercise profoundly impacts cortical bones that are refractory to current drugs. However, the mechanisms underlying these changes remain unclear. Here we show that mechanosensitive primary cilia on myeloid-lineage preosteoclasts have a critical role in mediating exercise-induced periosteal-bone formation. We crossed *Cx3cr1-Cre* or *Ctsk-Cre* mice with Ift88^*loxP/loxP*^ and/or Kif3a^*loxP/loxP*^ mice to create models that lack primary cilia on myeloid-lineage cells at various stages of osteoclastic differentiation. On exercise, all these mice displayed decreases in periosteal-bone formation and cortical-bone size. Mechanical stimulation of primary cilia on preosteoclasts activated Dvl2, suppressing cathepsin K production and preventing periostin degradation. The resulting elevation of periostin levels promoted periosteal-bone formation. Notably, cathepsin K inhibition overcame the effects of primary cilia deficiency in exercised Cx3cr1-Cre;Ift88^*loxP/loxP*^ mice, restoring cortical-bone size, bone formation, and periosteal periostin levels to those observed in exercised Ift88^*loxP/loxP*^ littermates. By uncovering the primary cilia–preosteoclast–Ctsk–periostin axis, this study provides a foundation for developing targeted therapies to enhance cortical-bone strength.

## Primary cilia drive exercise benefits for cortical-bone health

The study explores the role of primary cilia on myeloid-lineage cells in bone physiology, particularly in response to mechanical loading such as exercise. It reveals that primary cilia on preosteoclasts, a type of myeloid-lineage cell, are crucial for sensing mechanical stimuli and promoting cortical-bone growth. The researchers used genetically modified mice to demonstrate that exercise-induced activation of primary cilia on preosteoclasts reduces the production of cathepsin K (Ctsk), an enzyme that degrades periostin, a protein essential for bone formation. This reduction in Ctsk leads to increased periostin levels, enhancing osteoblast activity and cortical-bone size. The findings suggest that targeting the primary cilia–preosteoclast–Ctsk–periostin–osteoblast axis could improve bone strength and reduce fracture risk. Future research could explore the potential for therapeutic interventions that mimic or enhance this pathway to treat bone fragility. This summary was initially drafted using artificial intelligence, then revised and fact-checked by the author.

## Introduction

Owing to population aging worldwide, fragility fractures have become a serious socioeconomic issue. Drugs have been developed to ameliorate skeletal fragility, greatly reducing fracture risk in vertebral bones, but they have limited effects on non-vertebral bones^1^. This reflects the fact that most research has focussed on trabecular bone (which predominates in vertebrae), not on cortical bone (which predominates in non-vertebral bones)^2^. However, cortical bone is now known to contribute more to bone strength than trabecular bone^3^. This is also important because non-vertebral fractures account for 70–80% of all fragility fractures^4^. Thus, to improve skeletal-fragility management, more research on cortical bone is needed.

It is well known that mechanical loading, imposed by exercise, physical activity, and muscular contraction, increases bone strength. Interestingly, it primarily affects cortical bone rather than trabecular bone: a systematic review found that exercise promotes cortical-bone mass, area, and/or strength but has less potent effects on trabecular bone^5^. Similarly, a randomized-controlled trial reported that high-impact exercise substantially increases cortical, but not trabecular, bone mass^6^. Thus, mechanical loading, especially that imposed by exercise, is one of the best ways to study cortical-bone physiology. Notably, mechanical loading does not just increase cortical-bone mass: studies show that even in the absence of increased bone mass^7–12^, exercise and physical activity increase cortical-bone size and periosteal perimeter, both of which are primarily shaped by periosteal-bone formation^2,13^. As resistance to bending increases to the fourth power of the radius of cortical bone, even a small difference in the periosteal perimeter can contribute substantially to strength and resistance to fracture^4^. However, the detailed molecular mechanisms by which mechanical loading shapes cortical-bone size and periosteal-bone formation remain unclear.

Myeloid-lineage cells at bone include tartrate-resistant acid phosphatase (TRAP)-negative macrophages, TRAP+ mononuclear preosteoclasts, and TRAP+ multinuclear osteoclasts: the latter two are primarily known for their roles in bone resorption. It was long thought that osteoblast-lineage cells, including the highly abundant osteocytes, have a central role in the mechanosensitivity of the skeleton^14^, whereas the myeloid-lineage cells do not because they were thought to lack mechanosensing organelles. However, several recent studies together suggest that myeloid-lineage cells may also be mechanosensitive, and that this mechanosensitivity could shape cortical bone. First, two studies in mice undergoing normal mechanical loading from their daily activities showed that knockout (KO) of macrophage colony-stimulating factor not only depletes periosteal myeloid-lineage cells but also decreases periosteal cortical-bone formation. Both studies suggested that TRAP+ preosteoclasts were the key mediators of this effect^15,16^. Second, Deng et al.^17^ reported recently that periosteal myeloid-lineage cells increase cortical-bone formation by sensing mechanical loading. However, the study used a supraphysiological mechanical stimulus (periodic axial tibial compressive loading) and suggested that TRAP-negative macrophages, not TRAP+ preosteoclasts, drove cortical-bone formation in this setting^17^. Thus, at present, it is unclear whether TRAP+ osteoclasts/preosteoclasts can respond to physiological mechanical stimuli and thereby shape cortical-bone size.

Primary cilia are highly conserved microtubule-based organelles that project from the cell surface into the extracellular environment and can sense mechanical signals^18^. The roles that primary cilia have in bone-cell functions have been primarily studied in osteoblast-lineage cells, including osteocytes. These studies show that these cells in both trabecular and cortical bones sense mechanical load^19^ and promote the osteogenic and chondrogenic induction of mesenchymal-stem cells^18^, bone development^20^, and osteoblast differentiation^21^. However, a very recent study reported that skin dendritic cells also bear primary cilia^22^, which suggests that bone myeloid-lineage cells may also be mechanosensitive. However, little is known about the roles that primary cilia on myeloid-lineage cells have in bone physiology. Our present study suggests that primary cilia on periosteal preosteoclasts are important contributors of the ability of exercise, which is a physiological mechanical force, to increase cortical-bone size. The molecular mechanisms underlying these roles were also elucidated.

## Materials and methods

### Animals and treatments

*Ctsk-cre* (MGI, 3764465), *Col2.3-cre* (Riken, RBRC05603), and *Cx3cr1-cre*/*ERT2* mice (Jackson Laboratory, no. 020940) were mated with Ift88^*loxP/loxP*^ (Jackson Laboratory, no. 022409) and/or Kif3a^*loxP/loxP*^ mice (MGI, no. 2386464). To induce gene KO, peritoneal injections of tamoxifen (75 mg/kg/day, Sigma-Aldrich) were administered to 11-week-old Cx3cr1;Ift88^*loxP/loxP*^ mice and their Ift88^*loxP/loxP*^ littermates for 5 days. To assess gene-KO efficiency, bone-marrow cells were harvested post-sacrifice and differentiated into preosteoclasts and osteoblasts for 3 and 21 days, respectively. Ift88 and Kif3a expression by the differentiated cells was confirmed by western blot. There were no skeletal deficits in the *Ctsk-cre*, *Col2.3-cre*, and *Cx3cr1-cre*/*ERT2* mice used in this study^23^.

To assess the effect of exercise, 12-week-old mice underwent exercise training on a 10° incline treadmill running at a speed of 15 m/min for 30 min, 5 times/week for 4 weeks. To assess the effect of exercise restriction (designated unloaded or Un), 14-week-old mice were exposed to hind-limb suspension via a tail-suspension device for 2 weeks. All mice were euthanized at 16 weeks of age. To assess the effect of HL235 treatment, 12-week-old Cx3cr1;Ift88^*loxP/loxP*^ mice received 100 μl HL235 (100 mg/kg) orally twice weekly for 4 weeks concomitant with exercise. Although the oral bioavailability of HL235 in rats is relatively low (3.87–6.78%)^24^, its therapeutic efficacy for the treatment of osteoporosis via oral administration has been previously demonstrated. Negative-control mice received the same dose of vehicle (8% DMSO, 10% ethanol, 30% PEG400, and 10% Cremophor EL in saline solution) orally. Before euthanasia, all mice were anesthetized with Zoletil (50 mg/kg, i.p.) and Xylazine (10 mg/kg, i.p.), and their organs were harvested for analysis. Randomization and blinding were applied in the HL235 treatment experiment.

All animal protocols were approved by the Institutional Animal Care and Use Committee of the Asan Institute for Life Sciences (Approval No. 2021-01-025). All animal studies followed the ARRIVE guidelines 2.0. Euthanasia procedures were performed according to the guidelines of the Institutional Animal Care and Use Committee of the Asan Institute of Life Sciences.

For additional materials and methods, see Supplementary information.

## Results

### The increased cortical-bone size induced by exercise is mediated by primary cilia on myeloid-lineage cells

**Fig. 1:**
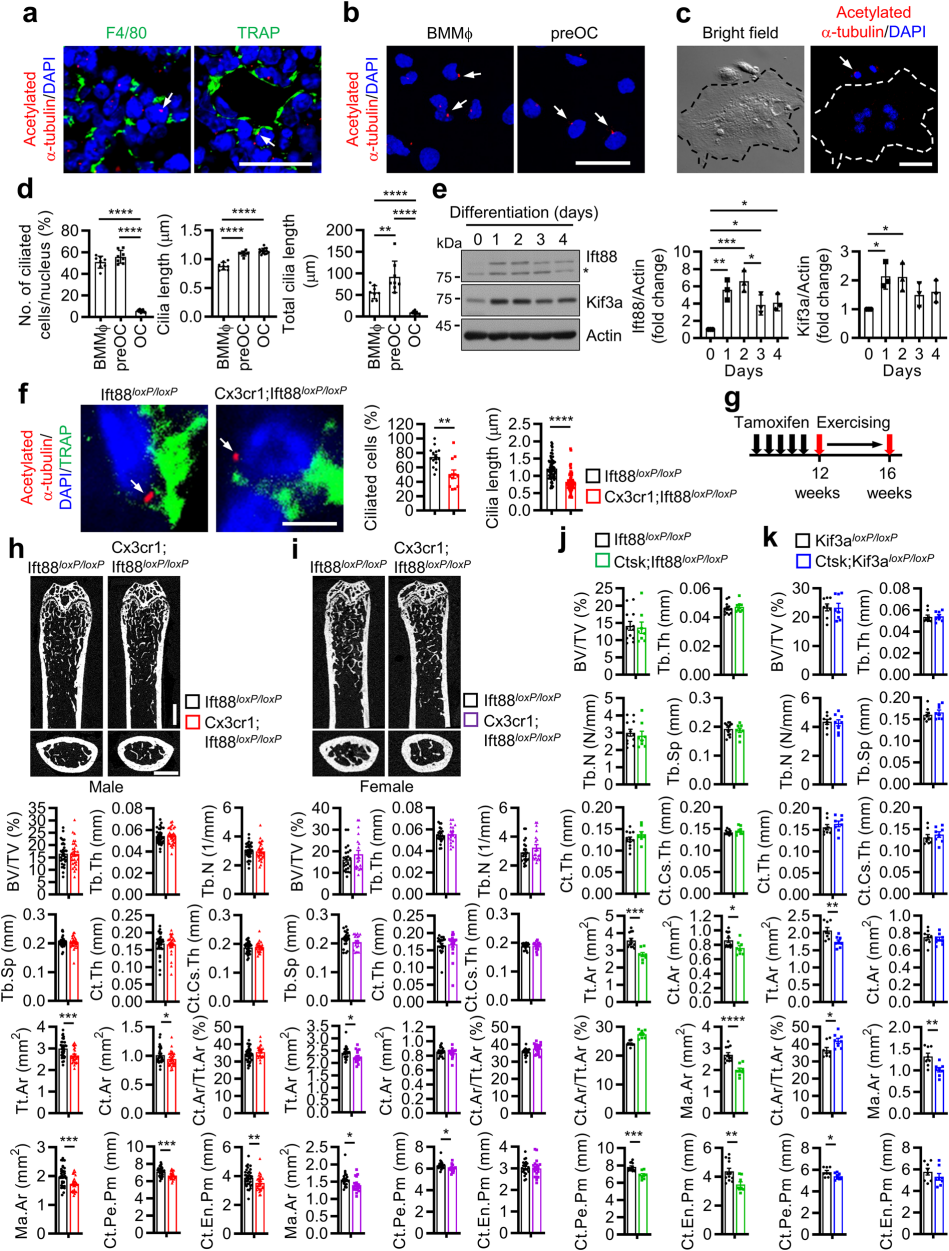
Exercise-induced stimulation of primary cilia on myeloid-lineage cells increases cortical-bone size. **a** Immunostaining of acetylated α-tubulin (red) and F4/80 (green) or tartrate-resistant acid phosphatase (TRAP) (green) in femurs of 16-week-old male C57BL/6 mice. Scale bar, 20 μm. Immunostaining of acetylated α-tubulin (red) in bone-marrow macrophages (BMMϕ), preosteoblasts (preOCs) (part **b**), and osteoclasts (OCs) (part **c**). Scale bar, 20 μm. **d** Frequency of ciliated cells, cilium lengths, and total-cilia length (no. of ciliated cells × each length) in BMMϕ (*n* = 8), preOCs (*n* = 8), and OCs (*n* = 14). **e** BMMϕ were treated with macrophage colony-stimulating factor and RANKL for 1–4 days to induce preOCs and OCs (see Materials and methods). Intraflagellar-transport-88 (Ift88) and kinesin-family member-3a (Kif3a) expressions were analyzed by western blot. *Nonspecific bands. Right: Ift88 and Kif3a band densities (all, *n* = 3). **f** Immunostaining of acetylated α-tubulin (red) and TRAP (green) at the femoral periosteum of 16-week-old Cx3cr1;Ift88^*loxP/loxP*^ (*n* = 12) and Ift88^*loxP/loxP*^ littermates (*n* = 15). Scale bar, 5 μm. Right: ciliated TRAP+ mononuclear-cell frequencies and cilia length (see Materials and methods). **g**–**i** Eleven-week-old male (part **h**) and female Cx3cr1;Ift88^*loxP/loxP*^ mice (part **i**) and Ift88^*loxP/loxP*^ littermates were administered tamoxifen for 5 days. Twelve-week-old mice underwent treadmill exercise for 4 weeks. **h**, **i**, Upper: representative micro-CT images of proximal femurs of 16-week-old Cx3cr1;Ift88^*loxP/loxP*^ mice (*n* = 32 (part **h**) and *n* = 20 (part **i**)) and Ift88^*loxP/loxP*^ littermates (*n* = 35 (part **h**) and *n* = 23 (part **i**)). Scale bar, 1 mm. **h**, **i** Lower: quantitative results. **j**, **k** Male Ctsk;Ift88^*loxP/loxP*^, Ctsk;Kif3a^*loxP/loxP*^, Ift88^*loxP/loxP*^, and Kif3a^*loxP/loxP*^ mice were exercised for 4 weeks. Quantitative micro-CT results of proximal femurs of Ctsk;Ift88^*loxP/loxP*^ (*n* = 8) and Ift88^*loxP/loxP*^ mice (*n* = 12) (part **j**) and Ctsk;Kif3a^*loxP/loxP*^ (*n* = 8) and Kif3a^*loxP/loxP*^ mice (*n* = 8) (part **k**). Mean ± SD (parts **d** and **e**) or mean ± SEM (parts **f**–**k**) is shown. Arrows indicate primary cilia (parts **a**–**c** and **f**). Statistical significance was determined by Student’s unpaired two-tailed *t* test (parts **f**–**k**) or one-way analysis of variance followed by Tukey’s test (parts **d** and **e**). *P < 0.05, **P < 0.01, ***P < 0.001, ****P < 0.0001. BV/TV, trabecular bone volume/tissue volume; Ct.Ar, cortical area; Ct.Cs.Th, cortical cross-sectional thickness; Ct.En.Pm, cortical-endosteal perimeter; Ct.Pe.Pm, cortical-periosteal perimeter; Ct.Th, cortical thickness; DAPI, 4ʹ,6-diamidino-2-phenylindole; Ma.Ar, marrow area; Tb.N, trabecular number; Tb.Sp, trabecular spacing; Tb.Th, trabecular thickness; Tt.Ar, total cross-sectional area inside periosteal envelope.

We first asked whether myeloid-lineage cells in bone bear primary cilia. We observed that bone-marrow-derived F4/80+ macrophages (BMMϕ), preosteoclasts, and osteoclasts bore these organelles (Fig. 1a–c and Supplementary Fig. 1a, b). However, osteoclasts were much less ciliated than BMMϕ and preosteoclasts, whereas preosteoclasts and osteoclasts had longer cilia than that for BMMϕ. Consequently, preosteoclasts had greater total cilia lengths (that is, cilia length per cell times the number of ciliated cells) than those of osteoclasts and BMMϕ (Fig. 1d). In human peripheral blood monocyte-derived osteoclast-lineage cells, although the number of primary cilia was not substantially different between BMMϕ and preosteoclasts, preosteoclasts exhibited substantially greater ciliary length than BMMϕ (Supplementary Fig. 1c, d). In contrast to murine osteoclasts, no detectable primary cilia were observed in mature human osteoclasts. Moreover, western blot of the primary-cilium components intraflagellar-transport-88 (Ift88) and kinesin-family member-3a (Kif3a) during in vitro differentiation of BMMϕ into osteoclasts showed that both were most strongly expressed in the early stages, which is when preosteoclasts were most abundant (Fig. 1e). Thus, myeloid-lineage cells, particularly preosteoclasts, bear primary cilia and could therefore potentially sense mechanical stimuli.

To determine the role of primary cilia on myeloid-lineage cells, we crossed Ift88^*loxP/loxP*^ mice with tamoxifen-inducible CX3C-motif chemokine receptor-1 (*Cx3cr1*)-*cre* mice (Supplementary Fig. 1e, f). Preosteoclasts generated from Cx3cr1;Ift88^*loxP/loxP*^ bone marrow were ciliated less often than Ift88^*loxP/loxP*^ littermate-control preosteoclasts and had shorter cilia. Confocal analysis of femurs confirmed that the conditional KO reduced the frequency of ciliated TRAP+ mononuclear cells and their cilia lengths in vivo (Fig. 1f). As expected, Cx3cr1;Ift88^*loxP/loxP*^ osteoblasts had normal *Ift88* expression, ciliation, and cilia lengths (Supplementary Fig. 1e–h).

Cx3cr1;Ift88^*loxP/loxP*^ and Ift88^*loxP/loxP*^ mice were then subjected to treadmill exercise (Fig. 1g) or, as a negative control, exercise restriction via hind-limb suspension (designated Unloading) (Supplementary Fig. 1i). Mice that allowed free physical activity did not serve as negative controls to eliminate the effects of minor exercise as much as possible. Micro-CT of Cx3cr1;Ift88^*loxP/loxP*^ and Ift88^*loxP/loxP*^ mice showed that they did not differ in femoral trabecular-bone variables, regardless of whether they had undergone exercise or hind-limb unloading, or whether they were male (Fig. 1h and Supplementary Fig. 1i) or female (Fig. 1i and Supplementary Fig. 1j). Conversely, male and female Cx3cr1;Ift88^*loxP/loxP*^ mice had substantially lower total cross-sectional area (Tt.Ar), marrow area (Ma.Ar), and cortical-periosteal perimeter (Ct.Pe.Pm) than Ift88^*loxP/loxP*^ littermates under exercising conditions (Fig. 1h, i) but not when exercise was restricted (Supplementary Fig. 1i, j).

We next examined the effect of depleting primary cilia on only osteoclast-lineage cells by generating cathepsin K (*Ctsk*)-*cre* mice that lacked *Itf88* or *Kif3a* expression. On exercise, Ctsk;Ift88^*loxP/loxP*^ (Fig. 1j and Supplementary Fig. 2a–c, g) and Ctsk;Kif3a^*loxP/loxP*^ (Fig. 1k and Supplementary Fig. 2d–f, h) mice had similar phenotypes to Cx3cr1;Ift88^*loxP/loxP*^ mice, namely, decreased cortical-bone size variables (Tt.Ar, Ma.Ar, and Ct.Pe.Pm) and no change in trabecular-bone variables. These similarities suggest that preosteoclasts/osteoclasts drive primary cilia-mediated exercise-induced cortical-bone growth.

Notably, Ctsk is expressed in both osteoclast-lineage and osteoblast-lineage cells^25^. However, similar to our observations in Cx3cr1;Ift88^*loxP/loxP*^ mice (Supplementary Fig. 1e–h), only preosteoclasts, not osteoblasts, demonstrated reduced ciliation and cilia lengths in Ctsk;Ift88^*loxP/loxP*^ and Ctsk;Kif3a^*loxP/loxP*^ mice (Supplementary Fig. 2a–f).

**Fig. 2:**
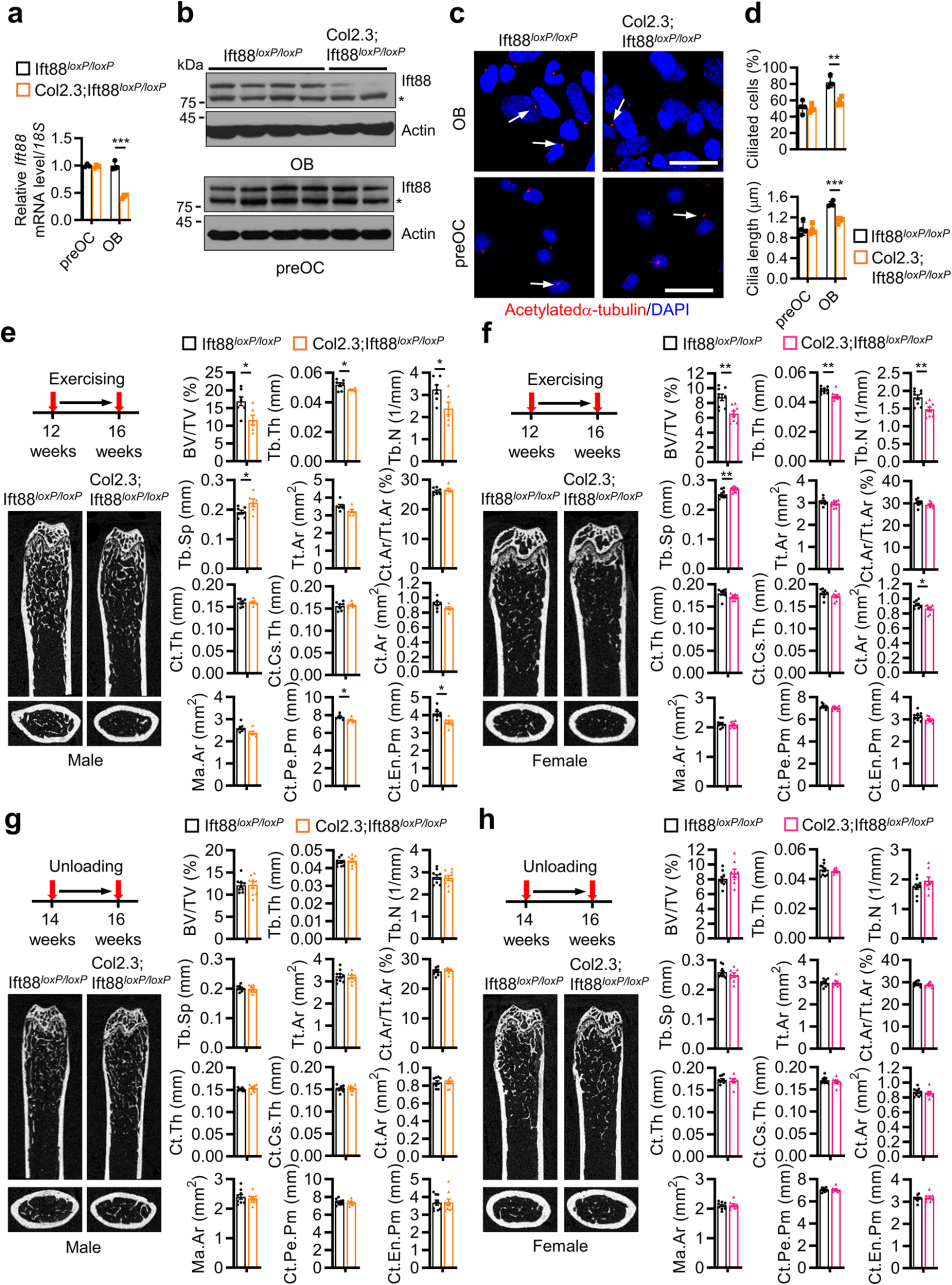
Micro-CT of femurs of mice whose osteoblast-lineage cells lack primary cilia. **a**–**d** Bone-marrow cells from 16-week-old male Col2.3;Ift88^*loxP/loxP*^ and Ift88^*loxP/loxP*^ mice were differentiated into osteoblasts (OBs) and preosteoclasts (preOCs) (see Materials and methods) and subjected to real-time PCR (*n* = 3) (part **a**) and western blot (part **b**). *Nonspecific bands. **c** Representative acetylated α-tubulin (red) immunostaining images of OBs and preOCs. Arrows, primary cilia. Scale bar, 20 μm. **d** Frequency of ciliated cells (upper) and cilia length (lower) (*n* = 4). Male (parts **e** and **g**) and female (parts **f** and **h**) Col2.3;Ift88^*loxP/loxP*^ and Ift88^*loxP/loxP*^ mice were exercised or suspended by their tails. Representative micro-CT images of the proximal femurs of male Col2.3;Ift88^*loxP/loxP*^ (*n* = 6 (part **e**) and *n* = 9 (part **g**)) and Ift88^*loxP/loxP*^ mice (*n* = 7 (part **e**) and *n* = 9 (part **g**)) and female Col2.3;Ift88^*loxP/loxP*^ (*n* = 9 (part **f**) and *n* = 8 (part **h**)) and Ift88^*loxP/loxP*^ mice (*n* = 9 (part **f**) and *n* = 10 (part **h**)). Quantitative results of micro-CT are shown on each right. Bone variables are detailed in Fig. 1. Mean ± SD (parts **a** and **d**) or mean ± SEM (parts **e**–**h**) is shown. Statistical significance was determined by two-way analysis of variance followed by Sidak’s post hoc test (parts **a** and **d**) or Student’s unpaired two-tailed *t* test (parts **e**–**h**). *P < 0.05, **P < 0.01, ***P < 0.001. BV/TV, trabecular bone volume/tissue volume; Ct.Ar, cortical area; Ct.Cs.Th, cortical cross-sectional thickness; Ct.En.Pm, cortical-endosteal perimeter; Ct.Pe.Pm, cortical-periosteal perimeter; Ct.Th, cortical thickness; DAPI, 4ʹ,6-diamidino-2-phenylindole; Ma.Ar, marrow area; Tb.N, trabecular number; Tb.Sp, trabecular spacing; Tb.Th, trabecular thickness; Tt.Ar, total cross-sectional area inside periosteal envelope.

Nonetheless, the potential involvement of osteoblasts in the *Ctsk* mutants led us to briefly examine the role of primary cilia on osteoblast-lineage cells. Thus, 2.3-kb type-I collagen promoter (*Col2.3*)*-cre* mice were crossed with Ift88^*loxP/loxP*^ mice. The osteoblast-lineage cells in the resulting Col2.3;Ift88^*loxP/loxP*^ mice had less ciliation and shorter cilia than their counterparts in Ift88^*loxP/loxP*^ littermate controls (Fig. 2a–d). On exercise, male (Fig. 2e) and female (Fig. 2f) Col2.3;Ift88^*loxP/loxP*^ mice had substantially lower trabecular-bone variables relative to Ift88^*loxP/loxP*^ littermates but most cortical-bone variables were comparable, although Ct.Pe.Pm and Ct.En.Pm were slightly lower in male Col2.3;Ift88^*loxP/loxP*^ mice. Under the unloaded condition, trabecular-bone and cortical-bone variables were unaltered in male and female Col2.3;Ift88^*loxP/loxP*^ mice relative to Ift88^*loxP/loxP*^ littermate controls (Fig. 2g, h).

Thus, primary cilia on myeloid-lineage cells (particularly preosteoclasts) and osteoclast-lineage cells increase cortical-bone size in response to exercise while not affecting on trabecular-bone mass. By contrast, primary cilia on osteoblast-lineage cells mainly increase trabecular-bone mass in response to exercise.

### Primary cilia on myeloid-lineage cells at the periosteum, not trabecular bone or endosteum, mediate exercise-induced bone formation

**Fig. 3:**
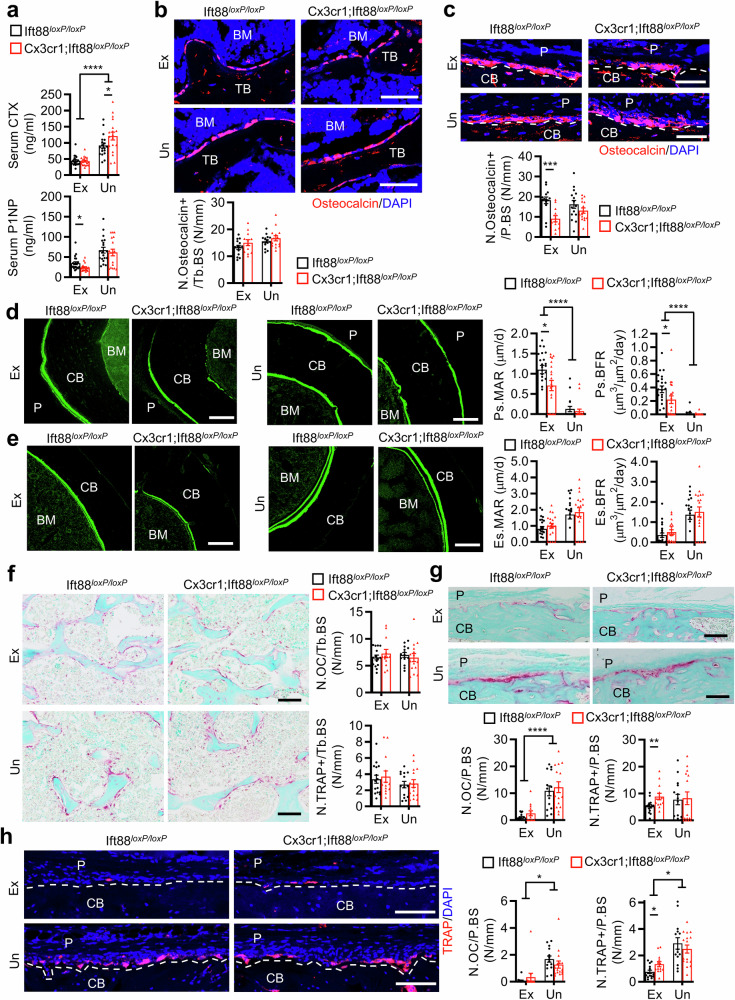
Exercised-stimulated primary cilia on myeloid-lineage cells promote periosteal-bone formation. **a**–**h** The indicated mouse strains were exercised (Ex) or suspended by their tails (Un) (Fig. 1). **a** Enzyme-linked immunosorbent assay-determined serum levels of C-terminal telopeptides of type-I collagen (CTX) and procollagen-1 N-terminal peptide (P1NP) in 16-week-old male Cx3cr1;Ift88^*loxP/loxP*^ (*n* = 21 Ex and *n* = 18 Un) and Ift88^*loxP/loxP*^ mice (*n* = 21 Ex and *n* = 19 Un). Representative osteocalcin (red) immunostaining images of femoral trabecular bone (part **b**) or periosteum (part **c**) in Cx3cr1;Ift88^*loxP/loxP*^ (part **b**, *n* = 12 Ex and *n* = 14 Un; part **c**, *n* = 12 Ex and *n* = 15 Un) and Ift88^*loxP/loxP*^ mice (parts **b** and **c**, *n* = 14 Ex and *n* = 15 Un). Scale bar, 50 μm. Bottom: quantification of osteocalcin+ numbers per trabecular-bone surface (N.Osteocalcin+/Tb.BS) and periosteal-bone surface (N.Osteocalcin+/P.BS). Representative calcin-staining images of femoral periosteal (Ps) (part **d**) and endocortical (Es) (part **e**) surfaces in Cx3cr1;Ift88^*loxP/loxP*^ (*n* = 20 Ex and *n* = 19 Un) and Ift88^*loxP/loxP*^ mice (*n* = 22 Ex and *n* = 17 Un). Scale bar, 100 μm. Right: mineral-apposition rate (MAR) and bone-formation rate (BFR) per periosteal surface (Ps.MAR and Ps.BFR) and endocortical surface (Es.MAR and Es.BFR). Representative tartrate-resistant acid phosphatase (TRAP) staining images of femoral trabecular (part **f**) and periosteal surfaces (part **g**) in Cx3cr1;Ift88^*loxP/loxP*^ (*n* = 13 Ex and *n* = 15 Un) and Ift88^*loxP/loxP*^ mice (*n* = 15 Ex and *n* = 14 Un). Scale bar, 100 μm. Part **f**, right; part **g**, below: osteoclast number per trabecular-bone surface (N.OC/Tb.BS) and periosteal-bone surface (N.OC/P.BS) and TRAP+ mononuclear-cell number per trabecular-bone surface (N.TRAP+/Tb.BS) and periosteal-bone surface (N.TRAP+/P.BS). **h** Representative TRAP (red) immunostaining images of femoral periosteal surfaces in Cx3cr1;Ift88^*loxP/loxP*^ mice (*n* = 13 Ex and *n* = 16 Un) and Ift88^*loxP/loxP*^ mice (*n* = 14 Ex and *n* = 14 Un). Scale bar, 100 μm. Right: N.OC/P.BS and N.TRAP+/P.BS. Mean ± SEM is shown. Statistical significance between Cx3cr1;Ift88^*loxP/loxP*^ and Ift88^*loxP/loxP*^ mice was determined with Student’s unpaired two-tailed *t* test. Statistical significance between Ex and Un groups was determined with two-way analysis of variance followed by Tukey’s test. *P < 0.05, **P < 0.01, ***P < 0.001, ****P < 0.0001. BM, bone marrow; CB, cortical bone; DAPI, 4ʹ,6-diamidino-2-phenylindole; P, periosteum; TB, trabecular bone.

To determine the effect of Cx3cr1;Ift88^*loxP/loxP*^ KO on global-bone resorption and formation, we measured serum levels of the bone-resorption marker C-telopeptide and the bone-formation marker procollagen type-1 N-terminal propeptide (P1NP). On exercise, Cx3cr1;Ift88^*loxP/loxP*^ mice had substantially lower P1NP levels than control mice but similar C-telopeptide levels. In the unloaded condition, Cx3cr1;Ift88^*loxP/loxP*^ KO slightly increased C-telopeptide levels (Fig. 3a). These data demonstrate that loss of cilia on myeloid-lineage cells abrogated the ability of exercise to increase cortical-bone size by decreasing bone formation, not by altering bone resorption.

Next, we examined the effect of cilia loss on myeloid-lineage cells on bone formation at the trabecular bone, periosteal surface, and endosteum. In response to exercise, Cx3cr1;Ift88^*loxP/loxP*^ mice had substantially fewer osteocalcin+ osteoblasts at the periosteum (Fig. 3c), but not at trabecular bones (Fig. 3b), than littermate controls. They also had lower periosteal-bone-formation rates (BFRs) and mineral-apposition rates (MAR) (Fig. 3d). By contrast, the KO and control mice had similar endosteal BFR and MAR, regardless of exercise status (Fig. 3e). Thus, exercise-stimulated primary cilia of myeloid-lineage cells specifically increase osteoblastic-bone formation at the periosteal surface, not at the endosteal surface or trabecular bone.

Exercised and unloaded Cx3cr1;Ift88^*loxP/loxP*^ and control mice had comparable osteoclast (N.OC) and TRAP-positive mononuclear-cell (N.TRAP+) numbers in trabecular bone (Fig. 3f). Exercise decreased numbers of osteoclasts (Fig. 3g, h) and F4/80-positive macrophages (Supplementary Fig. 3a) at the periosteum in both Cx3cr1;Ift88^*loxP/loxP*^ and control mice, but numbers of both cells were comparable between the Cx3cr1;Ift88^*loxP/loxP*^ and control mice. By contrast, two different methods showed that on exercise, Cx3cr1;Ift88^*loxP/loxP*^ mice had substantially more periosteal TRAP-positive mononuclear cells than control mice (Fig. 3g, h). These findings suggest that primary cilia in periosteal TRAP-positive mononuclear cells, rather than in periosteal osteoclasts or macrophages, may have a critical role in sensing exercise-induced mechanical stimuli. Moreover, the fact that exercised Cx3cr1;Ift88^*loxP/loxP*^ mice had more periosteal TRAP-positive mononuclear cells but less global-bone formation and fewer periosteal osteoblasts suggests that periosteal TRAP-positive mononuclear cells, including preosteoclasts, induce osteoblastic-bone formation in a cilia-dependent paracrine manner.

Both osteocytes and angiogenesis have key roles in regulating bone physiology: osteocytes sense and convert mechanical stimuli, thereby promoting bone formation^14^, whereas angiogenesis relates closely to osteogenesis^15^. However, we observed that regardless of exercise status, the trabecular and cortical bones of Cx3cr1;Ift88^*loxP/loxP*^ and Ift88^*loxP/loxP*^ mice had similar viable and dead osteocyte numbers (Supplementary Fig. 3b, c) and endomucin+ cell numbers (Supplementary Fig. 3d, e). These observations reveal that neither exercise nor loss of primary cilia on myeloid-lineage cells affected osteocyte numbers or angiogenesis.

### Shear-stressed primary cilia on preosteoclasts suppress their Ctsk production, thus reducing extracellular periostin degradation

**Fig. 4:**
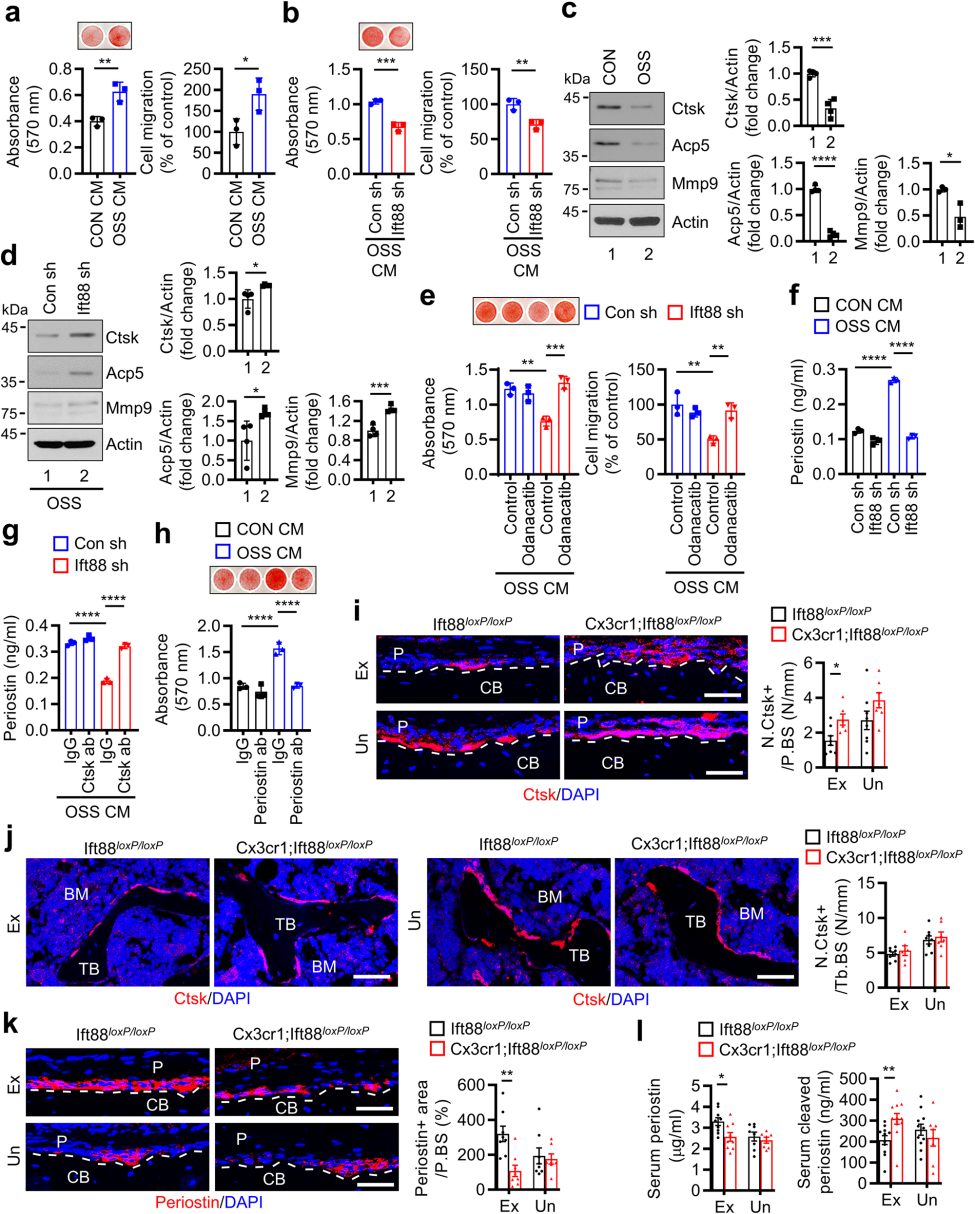
Shear-stressed primary cilia on preosteoclasts suppress their Ctsk secretion, thereby increasing local periostin levels. **a**, **b** Preosteoclasts were generated from bone-marrow macrophages (see Materials and methods) from wild-type mice and treated with orbital-shear stress (OSS) or cultured without it (CON) for 24 h. In part **b**, *Ift88* was knocked down before OSS. The conditioned medium (CM) was harvested. Calvarial-osteoblast precursors were then treated with CMs. **a** left: osteoblast mineralization after direct treatment with wild-type CM, as determined with Alizarin Red S staining (measured at 570 nm) (*n* = 3). **a** right: osteoblast migration was determined by placing osteoblasts in the upper chamber of a transwell and placing wild-type CM in the lower chamber. After 5 h, the migrated cells were stained, photographed, and counted (*n* = 3). **b** Effect of *Ift88* knockdown on CM-induced osteoblast-precursor mineralization (*n* = 3) and migration (*n* = 3). Western blots of cathepsin K (Ctsk), acid phosphatase-5 (Acp5), and matrix metallopeptidase-9 (Mmp9) in OSS-treated/CON-treated (part **c**) and wild-type and *Ift88*-knockdown (part **d**) preosteoclasts. Right: band densities (*n* = 4, except Mmp9 in part **c**, *n* = 3). **e** CM was obtained from wild-type and *Ift88*-knockdown preosteoclasts that were treated with 100 nM odanacatib and exposed to OSS for 24 h. CM-induced osteoblast-precursor mineralization (*n* = 3) and migration (*n* = 3) were measured. **f** Enzyme-linked immunosorbent assay (ELISA)-determined periostin levels in CM from OSS/CON-treated wild-type and *Ift88*-knockdown preosteoclasts (*n* = 3). **g** ELISA-determined periostin levels in CM from *Ift88*-knockdown preosteoclasts that were treated with Ctsk-blocking antibody (10 μg/ml) and OSS (*n* = 3). **h** Mineralization of osteoblast precursors incubated with OSS-treated wild-type preosteoclast CM that contained periostin-blocking antibody (5 μg/ml) (*n* = 3). **i**, **j** Male Cx3cr1;Ift88^*loxP/loxP*^ (*n* = 6 Ex and *n* = 7 Un) and Ift88^*loxP/loxP*^ mice (*n* = 8 Ex and *n* = 8 Un) were exercised (Ex) or suspended by their tails (Un) (Fig. 1). Representative Ctsk (red) immunostaining images at femoral periosteal (part **i**) and trabecular (part **j**) surfaces. Scale bar, 50 μm. Right: Ctsk+ number per periosteal (N.Ctsk+/P.BS) and trabecular (N.Ctsk+/Tb.BS) surfaces. **k** Representative periostin (red) immunostaining images in Cx3cr1;Ift88^*loxP/loxP*^ (*n* = 8 Ex and *n* = 7 Un) and Ift88^*loxP/loxP*^ mice (*n* = 8 Ex and *n* = 8 Un). Scale bar, 50 μm. Right: periostin+ area at periosteal-bone surface (Periostin+ area/P.BS). **l** ELISA-determined serum levels of periostin and cleaved periostin in male Cx3cr1;Ift88^*loxP/loxP*^ (periostin: *n* = 9 Ex and *n* = 7 Un; cleaved periostin: *n* = 12 Ex and *n* = 8 Un) and Ift88^*loxP/loxP*^ mice (periostin: *n* = 9 Ex and *n* = 8 Un; cleaved periostin: *n* = 11 Ex and *n* = 11 Un). Mean ± SEM (parts **i**–**l**) or mean ± SD (parts **a**–**h**) are shown. Statistical significance was determined by Student’s unpaired two-tailed *t* test (parts **a**–**d,** **i,** **k**, and **l**) or one-way analysis of variance followed by Tukey’s test (parts **e**–**h**). *P < 0.05, **P < 0.01, ***P < 0.001, ****P < 0.0001. BM, bone marrow; CB, cortical bone, DAPI, 4ʹ,6-diamidino-2-phenylindole; P, periosteum; TB, trabecular bone.

To test whether mechanically stressed primary cilia on preosteoclasts cause these cells to induce bone formation in a paracrine manner, we knocked down *Ift88* or *Kif3a* in preosteoclasts (Supplementary Fig. 4a), subjected the cells to orbital-shear stress (OSS) in vitro. We collected their conditioned medium (CM) and assessed its ability to induce osteoblastic activity. Indeed, CM from OSS-stimulated wild-type preosteoclasts induced osteoblast mineralization, migration (Fig. 4a), and alkaline phosphatase (ALP) activity (Supplementary Fig. 4b), and these effects were decreased by *Ift88* or *Kif3a* knockdown (Fig. 4b and Supplementary Fig. 4c).

However, our initial experiments could not identify the preosteoclast-derived paracrine factors responsible for bone formation: quantitative real-time PCR showed that multiple osteogenic candidates^15,17,26^ were not upregulated in preosteoclasts by OSS (Supplementary Fig. 4d) and that blocking their activity in the CM did not affect osteoblast ALP activity (Supplementary Fig. 4e, f). Therefore, we treated wild-type and *Ift88*-knocked down preosteoclasts with/without OSS and subjected them to RNA-sequencing analysis. Gene-set enrichment analysis showed that ossification and bone-remodeling gene sets were enriched not only by OSS of wild-type cells but also (in a converse manner) by *Ift88* knockdown in OSS-treated cells (Supplementary Fig. 4g, h). Of the top 5 ossification-related and bone remodeling-related genes that were most enriched, three were common to both conditions: *Ctsk*, acid phosphatase-5 (*Acp5*, which encodes TRAP), and matrix metallopeptidase-9 (*Mmp9*) (Supplementary Fig. 5a, b). Indeed, OSS suppressed all three genes in wild-type preosteoclasts (Fig. 4c and Supplementary Fig. 5c) and this suppression was alleviated by *Ift88* or *Kif3a* knockdown (Fig. 4d and Supplementary Fig. 5d, e). As TRAP is not a secreted factor, it was not assessed further. We next observed that OSS suppressed Ctsk-enzymatic activity in wild-type but not during *Ift88*/*Kif3a* suppression (Supplementary Fig. 5f). Moreover, when the Ctsk inhibitor odanacatib was added to the CM from OSS-treated *Ift88*/*Kif3a-*silencing preosteoclasts, they could now induce osteoblast mineralization, migration, and ALP activity (Fig. 4e and Supplementary Fig. 5g–j). However, an MMP9 inhibitor lacked this effect (Supplementary Fig. 5k). MMP9 was therefore not assessed further. Notably, when we harvested progenitor cells from the femoral periosteum, differentiated them into osteogenic-lineage cells in vitro^27^ (Supplementary Fig. 6a, b), and treated them with CM from OSS-treated wild-type and *Ift88*-knockdown preosteoclasts with/without odanacatib, the periosteum-derived osteoblasts showed the same responses as the bone marrow-derived osteoblasts (Supplementary Fig. 6c–e). These data suggest that mechanical stimulation activates primary cilia on preosteoclasts, which suppresses their release of Ctsk, promoting osteoblast bone-forming activities.

Ctsk directly cleaves and degrades periostin^28^, which has key roles in bone formation^29^. Notably, periostin is mainly expressed by the periosteum^30^. This production involves multiple cell types within the bone microenvironment, including osteoclast-lineage cells but also osteocytes, osteoblasts, and other periosteal cells^30^. We thus examined the role of periostin in the ability of activated cilia on preosteoclasts to promote osteoblast activity. First, we confirmed that recombinant Ctsk degraded recombinant periostin in both acidic and neutral pH(Supplementary Fig. 7a) and that this was reversed by Ctsk inhibition with a specific antibody or odanacatib (Supplementary Fig. 7b, c). Next, we found that OSS increased the periostin levels in CM from wild-type preosteoclasts but not *Ift88-*knockdown (Fig. 4f) and *Kif3a*-knockdown (Supplementary Fig. 7d) preosteoclasts. Importantly, inhibiting Ctsk increased the periostin levels in CMs from OSS-stimulated *Ift88*-knockdown and *Kif3a*-knockdown preosteoclasts (Fig. 4g and Supplementary Fig. 7e–g). Thus, preosteoclasts constitutively express periostin but it is largely degraded by the Ctsk that they concomitantly release. However, when mechanical stress activates primary cilia on preosteoclasts, these cells express less Ctsk, which alleviates local periostin degradation.

We next asked whether this preosteoclast–Ctsk–periostin axis shaped osteoblast activities. Consistent with previous studies^29,31^, we observed that adding recombinant periostin to osteoblasts enhanced their mineralization, migration, and ALP activity (Supplementary Fig. 7h–j). Moreover, treating a periostin-blocking antibody to CM from OSS-stimulated wild-type preosteoclasts abolished the ability of the CM to stimulate osteoblast activities (Fig. 4h and Supplementary Fig. 7k). These results demonstrate that when primary cilia on preosteoclasts are stressed, the cells produce less Ctsk, which reduces periostin degradation and increases osteoblastic-bone formation in vitro.

We next confirmed these findings in vivo. On exercise, Cx3cr1;Ift88^*loxP/loxP*^ mice had more Ctsk+ osteoclast-lineage cells at the periosteum (Fig. 4i), but not at the trabecular surface(Fig. 4j), and a smaller periostin+ area (Fig. 4k). Moreover, exercise in Ift88^*loxP/loxP*^ mice, but not Cx3cr1;Ift88^*loxP/loxP*^ mice, increased serum periostin levels while reducing serum levels of cleaved periostin (Fig. 4l).

**Fig. 5:**
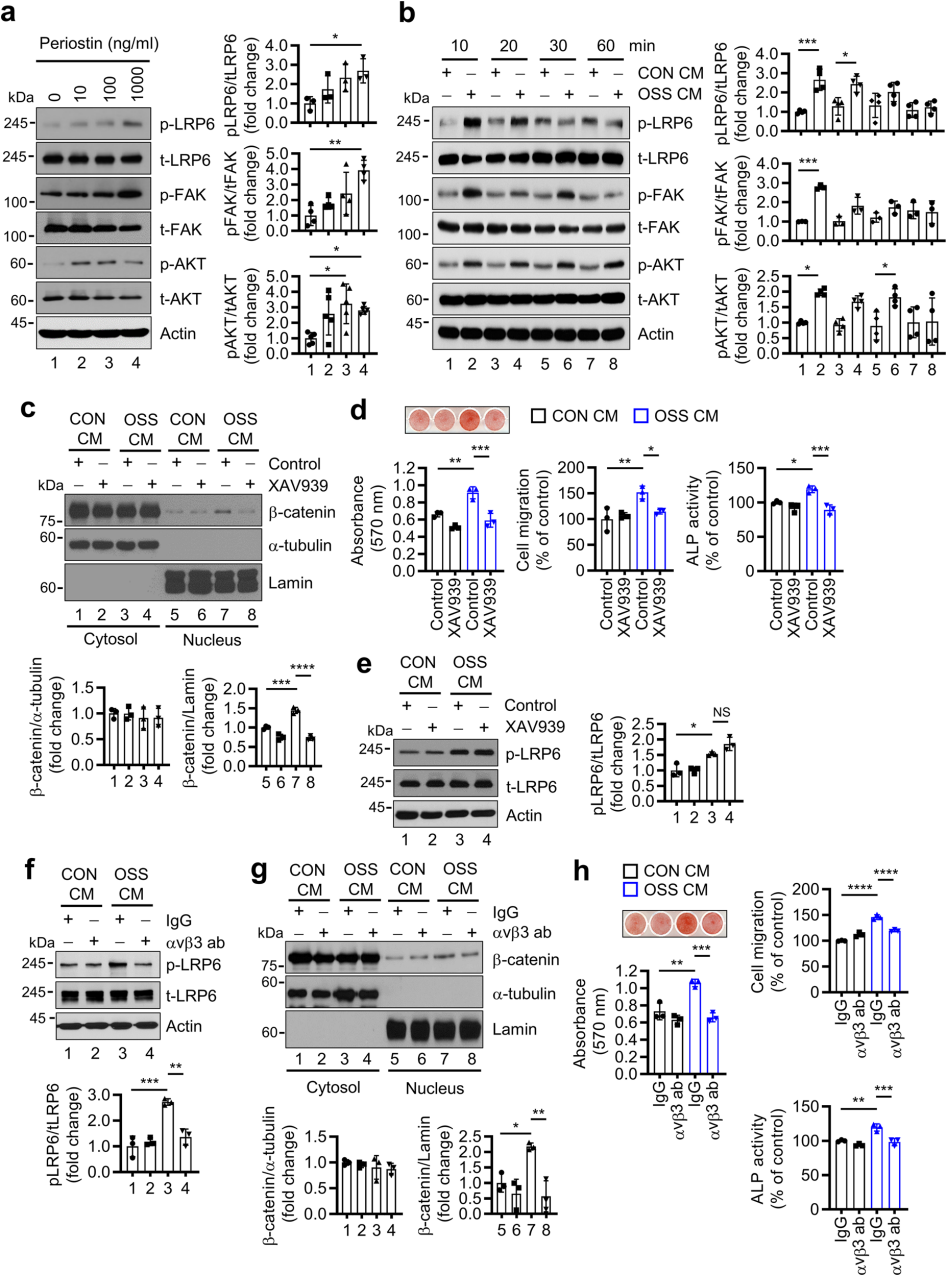
Secretions from shear-stressed preosteoclasts activate integrin-LRP6-β-catenin signaling in osteoblasts. **a**, **b** Calvarial-osteoblast precursors were treated with recombinant periostin at the indicated concentrations for 15 min (part **a**) or conditioned medium (CM) from preosteoclasts treated with (orbital-shear stress (OSS)) or cultured without (CON) OSS for 24 h (part **b**). Western blot was performed. Right: band densities (p-LRP6 *n* = 3, p-FAK *n* = 4, p-Akt *n* = 5 (part **a**); p-LRP6 and p-Akt *n* = 4, p-FAK *n* = 3 (part **b**)). **c**, **d** Osteoblast precursors were pretreated with/without 1 μM XAV939 and then with preosteoclast CM. **c** Western blot of their cytosolic and nuclear fractions. Bottom: band densities (*n* = 3). **d** Osteoblast mineralization, migration, and alkaline phosphatase (ALP) activity (all, *n* = 3). Osteoblast precursors were pretreated with 1 μM XAV939 (part **e**) or 10 μg/ml αvβ3-blocking antibody (part **f**) and then with preosteoclast CM for 15 min. **e** left; **f**, top: western blot was performed. **e**, right; **f** bottom: band densities (all, *n* = 3). **g**, **h** Osteoblast precursors were incubated with preosteoclast CM with/without integrin αvβ3-blocking antibody (10 μg/ml). **g** top: western blot of their cytosolic and nuclear fractions. **g**, bottom: band densities (*n* = 3). **h** Osteoblast mineralization (left), migration (top right), and ALP activity (bottom right) (all, *n* = 3). Mean ± SD is shown. One-way analysis of variance followed by Tukey’s test was performed. *P < 0.05, **P < 0.01, ***P < 0.001, ****P < 0.0001. FAK, focal-adhesion kinase; LPR, LDL receptor-related protein; NS, not significant.

The Wnt/β-catenin signaling pathway drives osteoblast differentiation and bone homeostasis and remodeling^32^. This pathway involves Wnt-ligand binding to the Wnt-ligand co-receptor LDLR-related protein-6 (LRP6), which activates the downstream pathway mediator Akt and focal-adhesion kinase (FAK)^33–35^. We found that treating osteoblasts with recombinant periostin in vitro activated their Wnt/β-catenin signaling pathway, as shown by phosphorylation of LRP6, Akt, and FAK (Fig. 5a). CM from OSS-stimulated wild-type preosteoclasts also increased LRP6, Akt, and FAK phosphorylation in osteoblasts (Fig. 5b). Moreover, both recombinant periostin and CM stimulated β-catenin nuclear translocation (Fig. 5c and Supplementary Fig. 8a). Adding XAV939, a Wnt inhibitor^36^, to the osteoblast cultures suppressed the ability of periostin (Supplementary Fig. 8b) and CM (Fig. 5d) to increase osteoblast mineralization, migration, and ALP activity. However, it did not affect the ability of periostin/CM to increase LRP6 phosphorylation (Fig. 5e and Supplementary Fig. 8c). Thus, both recombinant periostin and CM from OSS-stimulated wild-type preosteoclasts activate LRP6 and its downstream β-catenin in osteoblasts.

Periostin binds to the integrin-αvβ3 receptor^37^. As expected, its blocking antibody reversed the ability of periostin to promote Wnt signaling in osteoblasts and their bone-forming activities (Fig. 5f–h and Supplementary Fig. 8d–f).

### Primary-cilia activation reduces preosteoclast Ctsk expression by phosphorylating dishevelled-2 (Dvl2) and c-Fos degradation

**Fig. 6:**
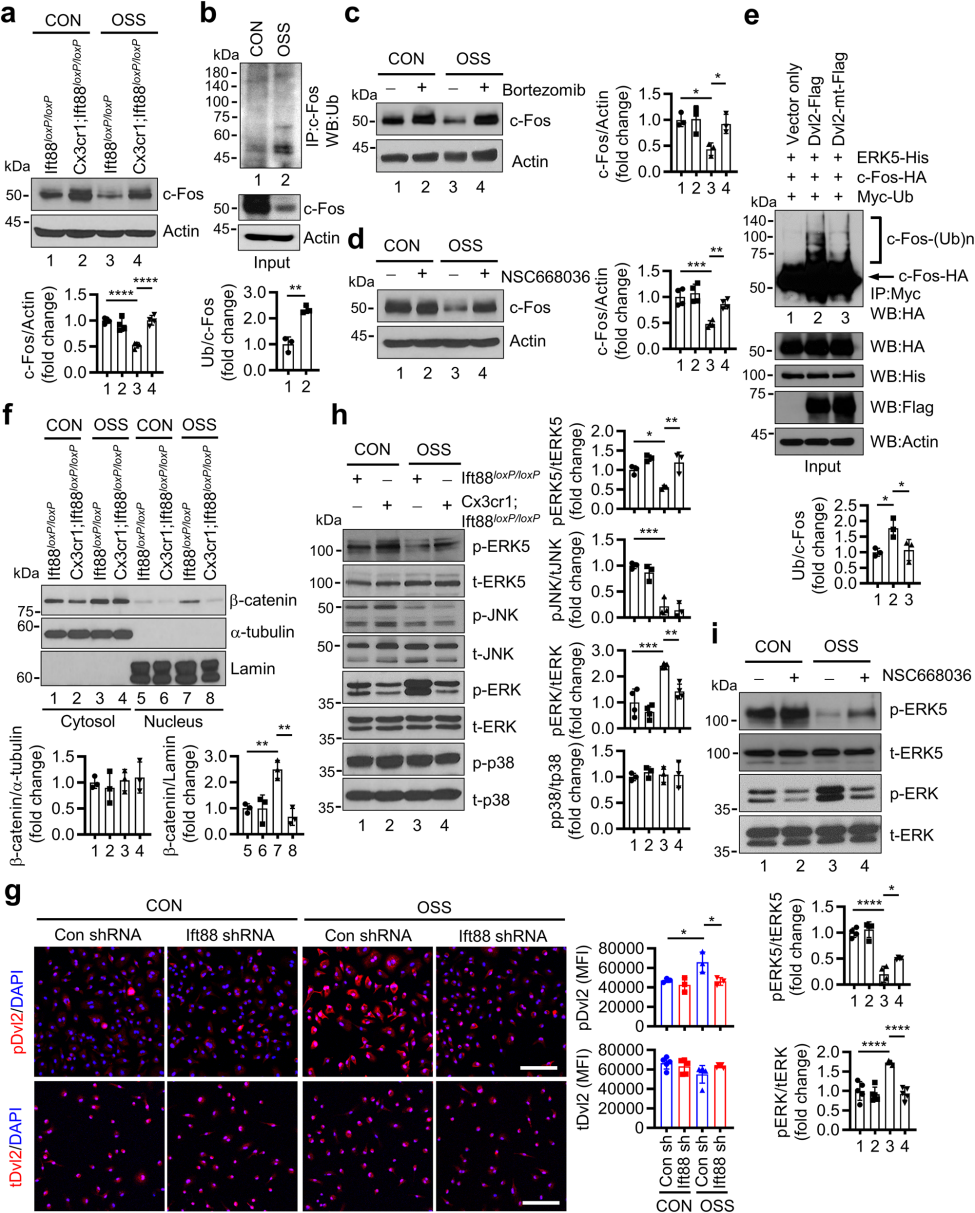
Shear-stressed primary cilia stimulate Dvl2 phosphorylation and c-Fos degradation in preosteoclasts. **a** Preosteoclasts were differentiated from bone-marrow macrophages from Cx3cr1;Ift88^*loxP/loxP*^ and Ift88^*loxP/loxP*^ mice (see Materials and methods), treated with orbital-shear stress (OSS)/cultured without (CON) OSS for 2 h, and subjected to western blot (WB) (*n* = 4). **b** Preosteoclasts treated with OSS and 200 nM MG132 for 8 h underwent immunoprecipitation (IP) with anti-c-Fos antibody and then WB analysis with anti-ubiquitin (Ub) antibody (*n* = 3). Preosteoclasts were pretreated with 100 nM bortezomib (part **c**) or 100 μM NSC668036 (part **d**) for 6 h, treated with OSS for 2 h, and analyzed by WB (part **c**, *n* = 3; part **d**, *n* = 4). **e** His-tagged ERK5-expressing HEK293T cells were transfected with HA-tagged c-Fos, Myc-tagged ubiquitin, Flag-tagged Dvl2, or Flag-tagged Dvl mutant (Dvl2-mt). Whole-cell extracts were immunoprecipitated with anti-myc antibody and analyzed by WB (*n* = 3). **f** Western blot of OSS-treated preosteoclast cytoplasmic and nuclear fractions (*n* = 3). **g** *Ift88*-knockdown preosteoclasts were treated with OSS for 30 min and phosphorylated Dvl2 (pDvl2) and total Dvl2 (tDvl2) were immunostained. Scale bar, 100 μm. Right: mean fluorescence intensity (MFI) of pDvl2 (*n* = 3, three independent) and tDvl2 (*n* = 5). **h** Preosteoclasts were treated with OSS for 30 min and analyzed by western blot (p-ERK *n* = 4; *n* = 3 for p-ERK5, p-JNK, and p-p38). **i**, Preosteoclasts were pretreated with 100 μM NSC668036 for 6 h, treated with OSS for 30 min, and analyzed by WB (p-ERK5 *n* = 4 and p-ERK *n* = 5). Band densities are shown below (parts **a**, **b**, **e**, **f**, and **i**) or to the right (parts **c**, **d**, and **h**). Mean ± SD is shown. Statistical significance was determined by Student’s two-tailed *t* test (part **b**) or one-way analysis of variance followed by Tukey’s test (parts **a** and **c**–**i**). *P < 0.05, **P < 0.01, ***P < 0.001, ****P < 0.0001. DAPI, 4ʹ,6-diamidino-2-phenylindole; shRNA, short hairpin RNA.

We next investigated the mechanism by which OSS regulates Ctsk expression in preosteoclasts. It is known that in osteoclast precursors, the c-Fos and c-Jun transcription factors bind to the *Ctsk* promoter to enhance its expression^38^. We found that steady-state wild-type and *Ift88*-silencing preosteoclasts expressed c-Fos protein, and OSS decreased c-Fos protein levels in wild-type but not in *Ift88*-deficient preosteoclasts (Fig. 6a and Supplementary Fig. 9a). However, neither OSS nor cilia deficiency affected c-Jun protein levels. As c-Fos protein levels are regulated by ubiquitination, which destabilizes c-Fos and targets it for proteasomal degradation^39^, we next assessed the effect of OSS on c-Fos ubiquitination. We found that OSS of wild-type preosteoclasts increased c-Fos ubiquitination (Fig. 6b). Moreover, the proteasome inhibitor bortezomib reversed the suppressive effect of OSS on c-Fos protein expression (Fig. 6c). Notably, OSS actually increased wild-type preosteoclast expression of c-*Fos* mRNA (Supplementary Fig. 9b), suggesting that OSS suppressed c-Fos protein expression in preosteoclasts by inducing c-Fos ubiquitination and proteasome degradation.

When primary cilia on vertebrate cells are stimulated by mechanical forces, they induce Wnt, Ca^2+^, and Hedgehog signaling^40,41^. However, treatment of wild-type preosteoclasts with inhibitors of each pathway showed that only the Wnt inhibitor NSC668036 (Fig. 6d), not the Ca^2+^ inhibitor BAPTA or the hedgehog inhibitor cyclopamine (Supplementary Fig. 9c, d), blocked OSS-suppressed c-Fos protein expression in preosteoclasts. Activation of Wnt signaling phosphorylates Dvl2. Our experiments showed that Dvl2 phosphorylation drives c-Fos ubiquitination: overexpressing Dvl2, but not the phosphorylation-resistant Dvl2 S143A mutant (Dvl2-mt), increased c-Fos ubiquitination (Fig. 6e). Moreover, we found that OSS increased not only nuclear β-catenin translocation but also Dvl2 phosphorylation in wild-type but not *Ift88*-deficient preosteoclasts (Fig. 6f, g and Supplementary Fig. 9e). These data indicate that OSS of primary cilia on preosteoclasts activates the Wnt pathway, leading to the phosphorylation of Dvl2. This event subsequently drives c-Fos protein ubiquitination and degradation, which downregulates Ctsk expression.

As shear stress can induce the phosphorylation of MAP kinases (MAPKs)^42,43^, and the Wnt pathway can crosstalk with MAPKs^44^, we examined the effect of OSS on the phosphorylation of various MAPKs in preosteoclasts. OSS, respectively, increased and decreased ERK5 and ERK phosphorylation in wild-type preosteoclasts, and this was blocked by *Ift88* knockdown (Fig. 6h and Supplementary Fig. 9f). The Wnt inhibitor NSC668036 at least partly prevented these OSS-induced changes in ERK5 and ERK (Fig. 6i). Thus, ERK5 and/or ERK may act as downstream mediators of the Wnt signaling pathway.

### Ctsk inhibition increases cortical-bone size in exercised mice that lack primary cilia on myeloid-lineage cells

**Fig. 7:**
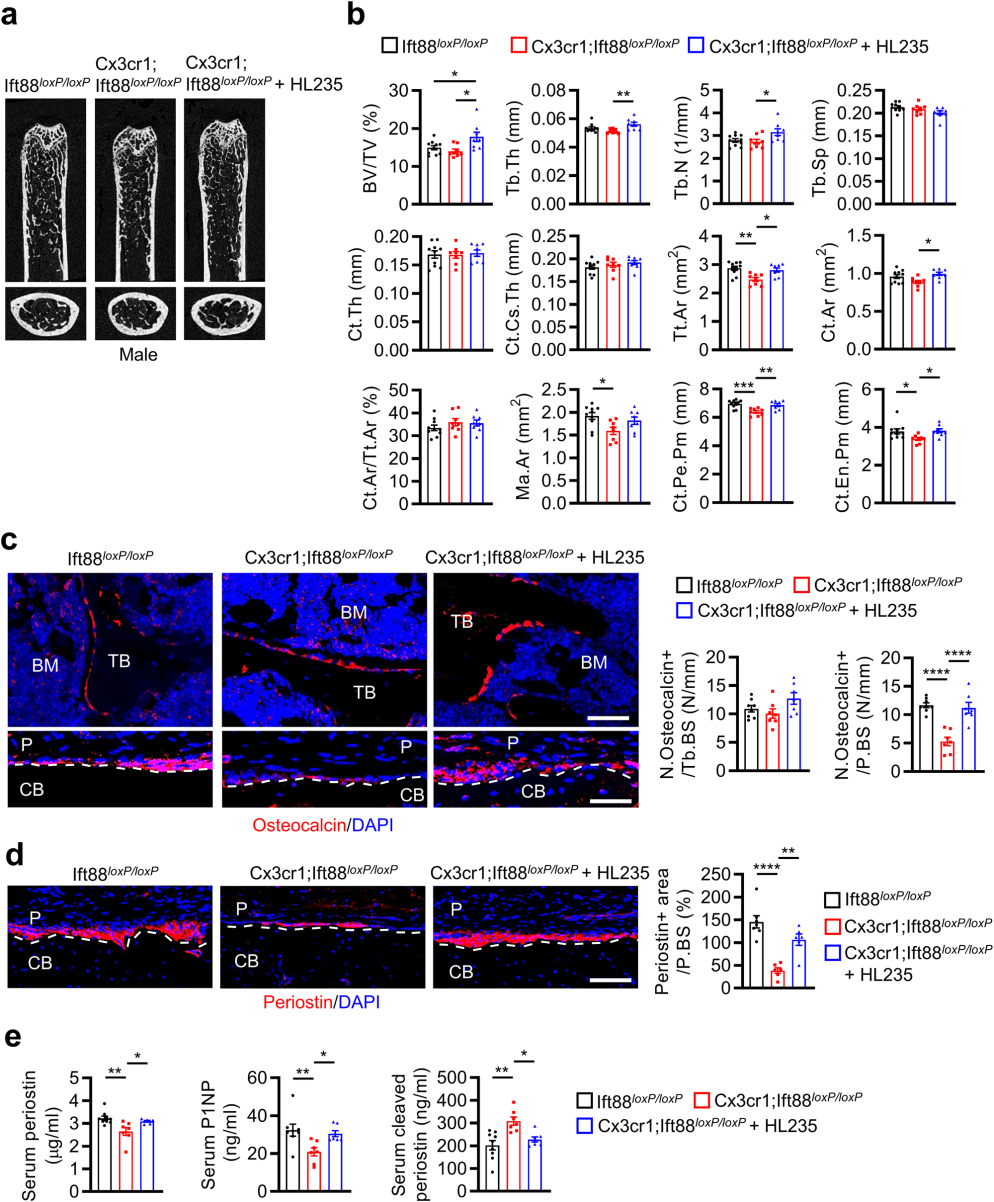
Inhibiting Ctsk increases cortical-bone size in exercised Cx3cr1;Ift88^*loxP/loxP*^ mice by elevating periostin levels. Male Cx3cr1;Ift88^*loxP/loxP*^ and Ift88^*loxP/loxP*^ littermates underwent treadmill exercise for 4 weeks (see Materials and methods). The Cx3cr1;Ift88^*loxP/loxP*^ mice were treated twice a week with/without oral 100 μl HL235 (100 mg/kg). **a** Representative micro-CT images of the proximal femurs of the exercised Ift88^*loxP/loxP*^ (*n* = 10), exercised Cx3cr1;Ift88^*loxP/loxP*^ (*n* = 8), and exercised HL235-treated Cx3cr1;Ift88^*loxP/loxP*^ mice (*n* = 8). **b** Quantitative results. Bone variables are described in Fig. 1. **c** Representative osteocalcin (red) immunostaining images of femurs of exercised Ift88^*loxP/loxP*^ (*n* = 8), exercised Cx3cr1;Ift88^*loxP/loxP*^ (*n* = 7), and exercised HL235-treated Cx3cr1;Ift88^*loxP/loxP*^ mice (*n* = 7). Scale bar, 50 μm. Osteocalcin+ number per trabecular-bone surface (N.Osteocalcin+/Tb.BS) and periosteal-bone surface (N.Osteocalcin+/P.BS) are shown in the right. **d** Representative periostin (red) immunostaining images of femurs in exercised Ift88^*loxP/loxP*^ (*n* = 7), exercised Cx3cr1;Ift88^*loxP/loxP*^ (*n* = 6), and exercised HL235-treated Cx3cr1;Ift88^*loxP/loxP*^ mice (*n* = 6). Scale bar, 100 μm. Right: periostin+ area at periosteal-bone surface (Periostin+ area/P.BS). **e** Enzyme-linked immunosorbent assay-determined serum levels of periostin (left), procollagen-1 N-terminal peptide (P1NP) (middle), and cleaved periostin (right) in exercised Ift88^*loxP/loxP*^ (periostin, cleaved periostin, *n* = 9; P1NP, *n* = 8), exercised Cx3cr1;Ift88^*loxP/loxP*^ (periostin, cleaved periostin, *n* = 7; P1NP, *n* = 7), and exercised HL235-treated Cx3cr1;Ift88^*loxP/loxP*^ mice (periostin, cleaved periostin, *n* = 7; P1NP, *n* = 7) mice. Mean ± SEM is shown. One-way analysis of variance was performed followed by Tukey’s test (parts **b**–**e**). *P < 0.05, **P < 0.01, ***P < 0.001, ****P < 0.0001. BM, bone marrow; BV, bone volume; CB, cortical bone; Ct.Ar, cortical area; Ct.Cs.Th, cortical cross-sectional thickness; Ct.En.Pm, cortical-endosteal perimeter; Ct.Pe.Pm, cortical-periosteal perimeter; Ct.Th, cortical thickness; DAPI, 4ʹ,6-diamidino-2-phenylindole; Ma.Ar, marrow area; P, periosteum; TB, trabecular bone; Tb.N, trabecular number; Tb.Sp, trabecular spacing; Tb.Th, trabecular thickness; Tt.Ar, total cross-sectional area inside periosteal envelope; TV, tissue volume.

As we showed that mechanical stimulation of primary cilia on preosteoclasts downregulates their production of Ctsk, which elevates local periostin levels and promotes osteoblast activity (Fig. 4), we asked whether inhibiting Ctsk could reverse the inability of mechanical loading to increase cortical bone in Cx3cr1;Ift88^*loxP/loxP*^ mice. To address this, these mice and their Ift88^*loxP/loxP*^ littermates underwent 4 weeks of exercise training while being concomitantly treated with/without the Ctsk inhibitor HL235 (ref. ^24^) twice weekly. HL235, similar to odanacatib, effectively blocks recombinant Ctsk activity, with IC_50_ values of 2.327 nM and 13.49 nM, respectively (Supplementary Fig. 10a). HL235 dose-dependently inhibited Ctsk activity without affecting other cathepsin isoforms (Supplementary Fig. 10b). Only Ctsk, but not other cathepsins, reduced periostin levels and cell migration, with selective reversals by HL235 (Supplementary Fig. 10c,d). HL235, similar to danacatib, effectively blocks recombinant Ctsk activity, with IC_50_ values of 2.327 nM and 13.49 nM, respectively (Supplementary Fig. 10a). HL235 dose-dependently inhibited Ctsk activity without affecting other cathepsin isoforms (Supplementary Fig. 10b). Importantly, Ctsk — but not other cathepsins — reduced periostin levels and cell migration, and these effects were selectively reversed by HL235 (Supplementary Fig. 10c, d). The cortical-bone loss in Cx3cr1;Ift88^*loxP/loxP*^ mice was completely reversed by HL235 treatment (Fig. 7a, b and Supplementary Fig. 10e, f). Concurrently, we found that HL235 treatment in exercised Cx3cr1;Ift88^*loxP/loxP*^ mice also substantially increased trabecular-bone mass compared with untreated exercised Ift88^*loxP/loxP*^ and Cx3cr1;Ift88^*loxP/loxP*^ mice in both sexes (Fig. 7a, b and Supplementary Fig. 10e, f). Therefore, blocking Ctsk activity sidestepped the lack of cilia on myeloid-lineage cells in Cx3cr1;Ift88^*loxP/loxP*^ mice, enabling these cells to promote cortical-bone formation.

We speculated that HL235 treatment increased trabecular bone by suppressing bone resorption: it is well known that inhibiting Ctsk increases bone mass in this manner^45^. By contrast, HL235 likely increased cortical bone via the primary cilia–preosteoclast–Ctsk–periostin–osteoblast mechanism we elucidated before. In this respect, we found that (i) HL235-treated exercised Cx3cr1;Ift88^*loxP/loxP*^, untreated exercised Ift88^*loxP/loxP*^, and untreated exercised Cx3cr1;Ift88^*loxP/loxP*^ mice had similar numbers of osteocalcin+ osteoblasts at trabecular bone (Fig. 7c); (ii) untreated exercised Cx3cr1;Ift88^*loxP/loxP*^ mice had fewer osteocalcin+ osteoblasts at the periosteum than untreated exercised Ift88^*loxP/loxP*^ littermates, as expected (Fig. 3c); and (iii) the latter was reversed by HL235 treatment (Fig. 7c). The same pattern was observed for periostin levels at the periosteal surface (Fig. 7d). It should be noted that periostin was mostly detected at the periosteum, not at trabecular bone (data not shown). This is consistent with previous reports^30^. The serum levels of periostin and the bone-formation marker P1NP showed the same patterns in the three mouse groups as the osteocalcin+ osteoblast numbers and periosteal periostin levels, whereas cleaved periostin levels in the serum revealed the converse pattern (Fig. 7e). Thus, Ctsk inhibition increased bone-forming cell numbers and bone formation by specifically increasing periostin levels at the periosteal surface in vivo. This observation confirms that the Ctsk–periostin axis acts downstream of the primary cilia on myeloid-lineage cells. By contrast, HL235 increases trabecular-bone mass by a different Ctsk-dependent mechanism that decreases bone resorption.

## Discussion

The skeleton recognizes and adapts to mechanical loading. In particular, repeated exercise increases cortical-bone strength, primarily by increasing cortical-bone size^5^. We showed here that this effect of exercise is mediated by mechanosensitive primary cilia on myeloid-lineage cells, particularly preosteoclasts, in the periosteum (Supplementary Fig. 10g). Specifically, the activated primary cilia decrease preosteoclast Ctsk production, which alleviates the degradation of extracellular periostin in the periosteum. The elevated periostin levels then activate osteoblasts, thereby increasing periosteal-bone formation and cortical-bone size. We also showed that treating exercised mice with primary cilia-deficient preosteoclasts with a Ctsk inhibitor increased their cortical-bone size to that seen in exercised control mice. The molecular mechanism involves exercise-stimulated primary-cilia activation of the canonical Wnt/β-catenin pathway in preosteoclasts: this induces Dvl2 phosphorylation, which promotes degradation of the c-Fos transcription factor, which then decreases preosteoclast Ctsk production and subsequently ameliorates periostin destruction in the periosteum. The now-abundant periostin binds to integrin-αvβ3 receptor on osteoblasts, which stimulates their LRP6–Akt-β–catenin axis and induces osteoblast migration, differentiation, and bone-forming activities. These activities only occur in the periosteum because it is the main producer of periostin. These findings provide evidence supporting that myeloid-lineage cells, particularly preosteoclasts, can sense physiological mechanical stimuli and are important contributors of exercise-induced periosteal-bone formation and cortical-bone size. Our study also suggests that targeting the primary cilia–preosteoclast–Ctsk–periostin–osteoblast axis could increase cortical-bone size.

These in vivo roles of primary cilia in myeloid-lineage cells were determined by generating male and female Cx3cr1;Ift88^*loxP/loxP*^, male Ctsk;Ift88^*loxP/loxP*^, and male Ctsk;Kif3a^*loxP/loxP*^ mice. Three variables were commonly decreased in all four of these animals on exercise, that is, Tt.Ar, Ct.Pe.Pm, and Ma.Ar., all of which relate to cortical-bone size. By contrast, trabecular-bone variables were not affected by the KO.

Bone myeloid-lineage cells include macrophages, preosteoclasts, and osteoclasts. We found that preosteoclasts bore longer cilia than macrophages and were ciliated much more often than osteoclasts. Moreover, the Cx3cr1;Ift88^*loxP/loxP*^ and Ctsk;Ift88^*loxP/loxP*^ lines had similar cortical-bone results but share only preosteoclasts and osteoclasts as mutation targets^25,46,47^. In addition, numbers of periosteal TRAP-positive mononuclear cells, but not those of periosteal osteoclasts, were altered in exercised Cx3cr1;Ift88^*loxP/loxP*^ mice. Thus, periosteal preosteoclasts are likely to be major exercise sensors.

In in vivo imaging studies, we cannot definitively determine whether the observed TRAP-positive mononuclear cells represent preosteoclasts or osteomorphs, owing to the lack of standardized and reliable methods to clearly distinguish these cell types in vivo^48^. Although we focussed on the role of preosteoclasts in vitro because these cells exhibited abundant primary cilia (Fig. 1d), we cannot exclude the possibility that osteomorphs may also act as exercise-sensing cells in vivo.

It should be noted that osteoblasts also express *Ctsk*^25^, albeit much less strongly than preosteoclasts and osteoclasts^49^. However, the potential effect of Ctsk;Ift88^*loxP/loxP*^ on osteoblasts likely did not shape outcomes: the osteoblast-lineage cells in Ctsk;Ift88^*loxP/loxP*^ and Ctsk;Kif3a^*loxP/loxP*^ mice had intact primary cilia, and we showed with Col2.3;Ift88^*loxP/loxP*^ mice that primary-cilia deficiency in osteoblast-lineage cells did not alter cortical-bone size. As animal models that lack primary cilia specifically on preosteoclasts are currently not available, we cannot verify this exercise-sensing role of preosteoblasts. Nonetheless, our data suggest for the first time that preosteoclasts are not just intermediate cells in osteoclast differentiation but also transmit mechanical stimuli and directly participate in bone metabolism.

Deng et al.^17^ recently suggested that early changes in periosteal macrophages, not periosteal preosteoclasts, led to loading-induced cortical-bone growth. It seems possible that both periosteal macrophages and preosteoclasts can contribute to mechanical loading-induced cortical-bone growth, albeit via different mechanical-signaling mechanisms (Piezo1 and primary cilia, respectively): neither our study nor theirs discounts this possibility. However, it should also be noted that we applied physiological stimuli (exercise), whereas Deng et al.^17^ applied a supraphysiological load (up to 12 N). Supraphysiological and physiological mechanical stimuli have different effects on the skeleton^50^. Nevertheless, this does not mean that physiological mechanical stimuli are sensed exclusively by periosteal preosteoclasts, as mechanical cues within the periosteal niche are likely detected by multiple mechanosensory systems. These mechanosensory pathways activate distinct downstream signaling programs, with Piezo1 promoting enhanced secretion and activation of transforming growth factor-β1 in macrophages and primary cilia inducing phosphorylation of Dvl2 in preosteoclasts. Thus, these pathways may function in parallel and/or interact to integrate heterogeneous mechanical inputs during periosteal-bone formation, rather than acting in a mutually exclusive manner.

Interestingly, our Col2.3;Ift88^*loxP/loxP*^ mouse analyses in both sexes showed that although primary-cilia deficiency on osteoblast-lineage cells did not affect exercise-induced cortical-bone growth, it did reduce trabecular-bone mass in exercised mice. These findings contrast with two other studies, which showed that osteoblast-lineage-specific primary-cilia KO decreased mechanical loading-induced periosteal-bone formation at the ulna^20,51^. However, both studies used supraphysiological mechanical stimulation (3.0/3.4 N loading)^52^, whereas we used physiological loading. Indeed, other studies on mature mice that were allowed to walk freely for ≥3 months showed that primary-cilia deficiency on osteoblast-lineage cells mainly decreased trabecular-bone mass^21,51^. Thus, primary cilia on preosteoclasts and osteoblast-lineage cells may, respectively, regulate cortical-bone size and trabecular-bone mass in response to physiological mechanical stimuli.

As the myeloid-lineage cells in bone include osteoclasts, this lineage is best known for its involvement in bone resorption^53^. Consequently, it was unexpected that exercised Cx3cr1;Ift88^*loxP/loxP*^ mice showed normal bone resorption (indicated by serum C-telopeptide) but decreased bone formation (indicated by serum P1NP). Bone analyses then showed that the decreased bone formation only occurred at the periosteum (indicated by decreased osteoblast numbers and BFR): it was not observed at the endocortical or trabecular surfaces. As this also associated with increased periosteal preosteoclast numbers, it suggested that periosteal preosteoclasts may inhibit osteoblastic bone formation via a paracrine mechanism. Indeed, our subsequent analyses then showed that this mechanism was mediated by preosteoclast-secreted Ctsk.

As primary-cilia deficiency in preosteoclasts did not affect serum C-telopeptide in exercised mice, we did not examine its role in bone resorption in this study. However, OSS decreased three bone-resorption proteins (*Ctsk*, *Mmp9*, and *Acp5*) in wild-type preosteoclasts, and this suppression was abolished by primary-cilia deficiency. Moreover, we observed that unloaded Cx3cr1;Ift88^*loxP/loxP*^ mice had higher serum C-telopeptide levels than unloaded Ift88^*loxP/loxP*^ littermates. As chemical stimuli also activate primary cilia^18^, it is possible that chemically stimulated primary cilia of myeloid-lineage cells can suppress bone resorption. Research on such mechanical stimuli-independent roles of primary cilia in bone myeloid-lineage cells is warranted.

The fact that exercised Cx3cr1;Ift88^*loxP/loxP*^ mice exhibited decreased osteoblast numbers and BFRs at only the periosteum likely reflects the primary cilia–preosteoclast–Ctsk–periostin–osteoblast axis we discovered and the fact that periostin is only abundant at the periosteum^30^. Notably, we also observed that in vitro*-*generated preosteoclasts constitutively express periostin, which suggests that these cells contribute to the periostin in the periosteum. However, other cells also likely generate this periostin, including osteocytes, osteoblasts, and other periosteal cells^29–31^. The mechanism by which periostin promotes osteoblast activity remains to be fully elucidated but Gao et al.^16^, and others^28^ suggest that it promotes the differentiation and adhesion of osteoblast progenitors. We also observed that recombinant periostin promoted the mineralization and migration of periosteum-derived osteoblast progenitor cells.

An important limitation of this study is the lack of direct in vivo validation of periostin function. Although genetic approaches, such as crossing periostin-deficient mice with primary cilia-deficient or *Ctsk*-overexpressing models, would provide more definitive evidence, these experiments were beyond the scope of the present work. Moreover, given the highly site-specific enrichment of periostin in the periosteum, it is currently not technically feasible to selectively neutralize periostin at the periosteal surface or to reliably distinguish and quantify cleaved versus intact periostin in vivo. Accordingly, a causal role for periostin in vivo remains to be established. Nevertheless, prior studies demonstrating that load-induced periosteal-bone formation and cortical thickening are markedly attenuated in periostin-deficient mice^54^ and are regulated by *Ctsk*^28^ strongly support the physiological relevance of the periostin–*Ctsk* axis, lending in vivo credibility to our proposed model. In addition, the present study used a single, standardized exercise/loading regimen rather than a systematic dose–response analysis of loading intensity or duration. Cortical bone is known to respond to mechanical loading in a dose-dependent manner^55^. Future studies incorporating dose–response analyses of exercise intensity and/or duration will be important to further enhance the translational relevance of exercise-based interventions for bone health.

Our study showed that mechanically stimulated preosteoclasts drive periosteal–osteoblastic function by decreasing their Ctsk production, and this is mediated by primary cilium-driven Wnt signaling that activates Dvl2 and β-catenin, thereby increasing c-Fos ubiquitination. These findings are consistent with previous studies, showing that (i) c-Fos modulates Ctsk-promoter activity^38^ and (ii) the canonical Wnt pathway is activated by primary-cilia stimulation^40^. We also observed that shear stress, respectively, suppressed and stimulated ERK5 and ERK phosphorylation in preosteoclasts in a primary cilia-dependent manner and that this was partially reversed by a Wnt inhibitor. This is supported by previous studies showing that shear stress regulates MAPK activities^42,43^. As ERK5 promotes c-Fos expression and stability, and the MEK inhibitor PD98059 increases c-Fos degradation^56^, it is likely that the primary cilia-stimulated canonical Wnt pathway partly increases c-Fos degradation by inhibiting ERK5.

We found that treating exercised Cx3cr1;Ift88^*loxP/loxP*^ mice with a Ctsk inhibitor not only increased their cortical-bone size to that seen in exercised Ift88^*loxP/loxP*^ mice, it also increased trabecular-bone mass. The increased trabecular-bone mass is likely due to the well-known antiresorptive activities of the Ctsk inhibitor as the inhibitor did not alter osteocalcin+ cell numbers at trabecular bone. By contrast, the inhibitor increased the periostin levels and osteocalcin+ cell numbers at the periosteum; however, these findings should be interpreted with caution, as the observed skeletal effects may, at least in part, reflect a well-established antiresorptive mechanism rather than selective modulation of the proposed primary cilia–preosteoclast axis. In addition, unlike murine osteoclasts, we did not detect primary cilia in mature human osteoclasts. This finding raises the possibility that the cilia-dependent mechanosensory pathway identified in this study may be species-specific or at least not fully conserved in humans. Although human preosteoclast-lineage cells showed a differentiation-dependent ciliary pattern, further studies will be required to validate whether this signaling mechanism is functionally operative in human cells and contributes to osteoclast-lineage mechanosensing in vivo.

In conclusion, myeloid-lineage cells, particularly preosteoclasts, sense mechanical stimuli via primary cilia, which increase cortical-bone size by decreasing preosteoclast Ctsk production and periostin degradation in the periosteum. Targeting this primary cilia–preosteoclast–Ctsk–periostin–osteoblast axis could potentially strengthen cortical bone, thereby reducing non-vertebral fracture risk.

## Supplementary information

Supplementary Information

## Data Availability

RNA-sequencing data were deposited in the Gene Expression Omnibus (GEO) under accession code GSE246066.

## References

[1] Lorentzon, M. Treating osteoporosis to prevent fractures: current concepts and future developments. *J Intern Med* **285**, 381–394 (2019). 30657216 10.1111/joim.12873

[2] Isojima, T. & Sims, N. A. Cortical bone development, maintenance and porosity: genetic alterations in humans and mice influencing chondrocytes, osteoclasts, osteoblasts and osteocytes. *Cell Mol Life Sci* **78**, 5755–5773 (2021). 34196732 10.1007/s00018-021-03884-w PMC11073036

[3] Augat, P. & Schorlemmer, S. The role of cortical bone and its microstructure in bone strength. *Age Ageing* **35**, ii27–ii31 (2006). 16926200 10.1093/ageing/afl081

[4] Bala, Y., Zebaze, R. & Seeman, E. Role of cortical bone in bone fragility. *Curr Opin Rheumatol* **27**, 406–413 (2015). 26002033 10.1097/BOR.0000000000000183

[5] Hamilton, C. J., Swan, V. J. & Jamal, S. A. The effects of exercise and physical activity participation on bone mass and geometry in postmenopausal women: a systematic review of pQCT studies. *Osteoporos Int* **21**, 11–23 (2010). 19504035 10.1007/s00198-009-0967-1

[6] Allison, S. J. et al. The influence of high-impact exercise on cortical and trabecular bone mineral content and 3D distribution across the proximal femur in older men: a randomized controlled unilateral intervention. *J Bone Miner Res* **30**, 1709–1716 (2015). 25753495 10.1002/jbmr.2499

[7] Jarvinen, T. L. et al. Randomized controlled study of effects of sudden impact loading on rat femur. *J Bone Miner Res* **13**, 1475–1482 (1998). 9738521 10.1359/jbmr.1998.13.9.1475

[8] Nilsson, M., Sundh, D., Mellstrom, D. & Lorentzon, M. Current physical activity is independently associated with cortical bone size and bone strength in elderly Swedish women. *J Bone Miner Res* **32**, 473–485 (2017). 27676223 10.1002/jbmr.3006

[9] Ng, C. A. et al. Associations between physical activity and bone structure in older adults: does the use of self-reported versus objective assessments of physical activity influence the relationship? *Osteoporos Int* **31**, 493–503 (2020). 31720706 10.1007/s00198-019-05208-y

[10] Osborn, W. et al. Bone health, activity and sedentariness at age 11-12 years: cross-sectional Australian population-derived study. *Bone* **112**, 153–160 (2018). 29674127 10.1016/j.bone.2018.04.011

[11] Hannam, K. et al. Habitual levels of higher, but not medium or low, impact physical activity are positively related to lower limb bone strength in older women: findings from a population-based study using accelerometers to classify impact magnitude. *Osteoporos Int* **28**, 2813–2822 (2017). 27966105 10.1007/s00198-016-3863-5 PMC5624975

[12] Dumuid, D. et al. The “Goldilocks Day” for children’s skeletal health: compositional data analysis of 24-hour activity behaviors. *J Bone Miner Res* **35**, 2393–2403 (2020). 32730680 10.1002/jbmr.4143

[13] Seeman, E. The periosteum — a surface for all seasons. *Osteoporos Int* **18**, 123–128 (2007). 17180552 10.1007/s00198-006-0296-6

[14] Carina, V. et al. Bone’s response to mechanical loading in aging and osteoporosis: molecular mechanisms. *Calcif Tissue Int* **107**, 301–318 (2020). 32710266 10.1007/s00223-020-00724-0

[15] Xie, H. et al. PDGF-BB secreted by preosteoclasts induces angiogenesis during coupling with osteogenesis. *Nat Med* **20**, 1270–1278 (2014). 25282358 10.1038/nm.3668 PMC4224644

[16] Gao, B. et al. Macrophage-lineage TRAP+ cells recruit periosteum-derived cells for periosteal osteogenesis and regeneration. *J Clin Invest* **129**, 2578–2594 (2019). 30946695 10.1172/JCI98857 PMC6538344

[17] Deng, R. et al. Periosteal CD68(+) F4/80(+) macrophages are mechanosensitive for cortical bone formation by secretion and activation of TGF-beta1. *Adv Sci (Weinh)* **9**, e2103343 (2022). 34854257 10.1002/advs.202103343 PMC8787385

[18] Li, X. et al. Role of primary cilia in skeletal disorders. *Stem Cells Int* **2022**, 6063423 (2022). 35761830 10.1155/2022/6063423 PMC9233574

[19] Verbruggen, S. W., Sittichokechaiwut, A. & Reilly, G. C. Osteocytes and primary cilia. *Curr Osteoporos Rep* **21**, 719–730 (2023). 37682373 10.1007/s11914-023-00819-1 PMC10724330

[20] Temiyasathit, S. et al. Mechanosensing by the primary cilium: deletion of Kif3A reduces bone formation due to loading. *PLoS ONE* **7**, e33368 (2012). 22428034 10.1371/journal.pone.0033368 PMC3299788

[21] Qiu, N. et al. Disruption of Kif3a in osteoblasts results in defective bone formation and osteopenia. *J Cell Sci* **125**, 1945–1957 (2012). 22357948 10.1242/jcs.095893 PMC3360919

[22] Toriyama, M. et al. Dendritic cell proliferation by primary cilium in atopic dermatitis. *Front Mol Biosci* **10**, 1149828 (2023). 37179569 10.3389/fmolb.2023.1149828 PMC10169737

[23] Elefteriou, F. & Yang, X. Genetic mouse models for bone studies — strengths and limitations. *Bone* **49**, 1242–1254 (2011). 21907838 10.1016/j.bone.2011.08.021 PMC3331798

[24] Visetvichaporn, V., Kim, K. H., Jung, K., Cho, Y. S. & Kim, D. D. Formulation of self-microemulsifying drug delivery system (SMEDDS) by D-optimal mixture design to enhance the oral bioavailability of a new cathepsin K inhibitor (HL235). *Int J Pharm* **573**, 118772 (2020). 31765770 10.1016/j.ijpharm.2019.118772

[25] Chai, W. et al. Visualizing cathepsin K-Cre expression at the single-cell level with GFP reporters. *JBMR Plus* **7**, e10706 (2023). 36699636 10.1002/jbm4.10706 PMC9850439

[26] McAllister, T. N., Du, T. & Frangos, J. A. Fluid shear stress stimulates prostaglandin and nitric oxide release in bone marrow-derived preosteoclast-like cells. *Biochem Biophys Res Commun* **270**, 643–648 (2000). 10753677 10.1006/bbrc.2000.2467

[27] Duchamp de Lageneste, O. et al. Periosteum contains skeletal stem cells with high bone regenerative potential controlled by Periostin. *Nat Commun* **9**, 773 (2018). 29472541 10.1038/s41467-018-03124-z PMC5823889

[28] Bonnet, N., Brun, J., Rousseau, J. C., Duong, L. T. & Ferrari, S. L. Cathepsin K controls cortical bone formation by degrading periostin. *J Bone Miner Res* **32**, 1432–1441 (2017). 28322464 10.1002/jbmr.3136

[29] Cobo, T. et al. Role of periostin in adhesion and migration of bone remodeling cells. *PLoS ONE* **11**, e0147837 (2016). 26809067 10.1371/journal.pone.0147837 PMC4725750

[30] Horiuchi, K. et al. Identification and characterization of a novel protein, periostin, with restricted expression to periosteum and periodontal ligament and increased expression by transforming growth factor beta. *J Bone Miner Res* **14**, 1239–1249 (1999). 10404027 10.1359/jbmr.1999.14.7.1239

[31] Liu, S. et al. Periostin regulates osteogenesis of mesenchymal stem cells from ovariectomized rats through actions on the ILK/Akt/GSK-3beta Axis. *Genet Mol Biol* **44**, e20200461 (2021). 34591063 10.1590/1678-4685-GMB-2020-0461 PMC8482812

[32] Vlashi, R., Zhang, X., Wu, M. & Chen, G. Wnt signaling: essential roles in osteoblast differentiation, bone metabolism and therapeutic implications for bone and skeletal disorders. *Genes Dis* **10**, 1291–1317 (2023). 37397540 10.1016/j.gendis.2022.07.011 PMC10311035

[33] Li, C., Williams, B. O., Cao, X. & Wan, M. LRP6 in mesenchymal stem cells is required for bone formation during bone growth and bone remodeling. *Bone Res* **2**, 14006 (2014). 26273519 10.1038/boneres.2014.6 PMC4472141

[34] Chen, L. L. et al. PI3K/AKT pathway involvement in the osteogenic effects of osteoclast culture supernatants on preosteoblast cells. *Tissue Eng A* **19**, 2226–2232 (2013). 10.1089/ten.tea.2012.0469 PMC3761438 23617625

[35] Salasznyk, R. M., Klees, R. F., Williams, W. A., Boskey, A. & Plopper, G. E. Focal adhesion kinase signaling pathways regulate the osteogenic differentiation of human mesenchymal stem cells. *Exp Cell Res* **313**, 22–37 (2007). 17081517 10.1016/j.yexcr.2006.09.013 PMC1780174

[36] Huang, S. M. et al. Tankyrase inhibition stabilizes axin and antagonizes Wnt signalling. *Nature* **461**, 614–620 (2009). 19759537 10.1038/nature08356

[37] Butcher, J. T., Norris, R. A., Hoffman, S., Mjaatvedt, C. H. & Markwald, R. R. Periostin promotes atrioventricular mesenchyme matrix invasion and remodeling mediated by integrin signaling through Rho/PI 3-kinase. *Dev Biol* **302**, 256–266 (2007). 17070513 10.1016/j.ydbio.2006.09.048 PMC1913192

[38] Pang, M., Martinez, A. F., Fernandez, I., Balkan, W. & Troen, B. R. AP-1 stimulates the cathepsin K promoter in RAW 264.7 cells. *Gene* **403**, 151–158 (2007). 17897792 10.1016/j.gene.2007.08.007

[39] Ito, Y., Inoue, D., Kido, S. & Matsumoto, T. c-Fos degradation by the ubiquitin–proteasome proteolytic pathway in osteoclast progenitors. *Bone* **37**, 842–849 (2005). 16172035 10.1016/j.bone.2005.04.030

[40] Anvarian, Z., Mykytyn, K., Mukhopadhyay, S., Pedersen, L. B. & Christensen, S. T. Cellular signalling by primary cilia in development, organ function and disease. *Nat Rev Nephrol* **15**, 199–219 (2019). 30733609 10.1038/s41581-019-0116-9 PMC6426138

[41] Shnitsar, I. et al. PTEN regulates cilia through Dishevelled. *Nat Commun* **6**, 8388 (2015). 26399523 10.1038/ncomms9388 PMC4598566

[42] Surapisitchat, J. et al. Fluid shear stress inhibits TNF-alpha activation of JNK but not ERK1/2 or p38 in human umbilical vein endothelial cells: inhibitory crosstalk among MAPK family members. *Proc Natl Acad Sci USA* **98**, 6476–6481 (2001). 11353829 10.1073/pnas.101134098 PMC33493

[43] Mengistu, M., Brotzman, H., Ghadiali, S. & Lowe-Krentz, L. Fluid shear stress-induced JNK activity leads to actin remodeling for cell alignment. *J Cell Physiol* **226**, 110–121 (2011). 20626006 10.1002/jcp.22311 PMC3290918

[44] Zhang, Y., Pizzute, T. & Pei, M. A review of crosstalk between MAPK and Wnt signals and its impact on cartilage regeneration. *Cell Tissue Res* **358**, 633–649 (2014). 25312291 10.1007/s00441-014-2010-x PMC4234693

[45] Pennypacker, B. L. et al. Inhibition of cathepsin K increases modeling-based bone formation, and improves cortical dimension and strength in adult ovariectomized monkeys. *J Bone Miner Res* **29**, 1847–1858 (2014). 24591096 10.1002/jbmr.2211

[46] Geissmann, F. et al. Development of monocytes, macrophages, and dendritic cells. *Science* **327**, 656–661 (2010). 20133564 10.1126/science.1178331 PMC2887389

[47] Fogg, D. K. et al. A clonogenic bone marrow progenitor specific for macrophages and dendritic cells. *Science* **311**, 83–87 (2006). 16322423 10.1126/science.1117729

[48] Park-Min, K. H., Mun, S. H., Bockman, R. & McDonald, M. M. New horizons: translational aspects of osteomorphs. *J Clin Endocrinol Metab* **109**, e1373–e1378 (2024). 38060842 10.1210/clinem/dgad711 PMC11031245

[49] Drake, M. T., Clarke, B. L., Oursler, M. J. & Khosla, S. Cathepsin K inhibitors for osteoporosis: biology, potential clinical utility, and lessons learned. *Endocr Rev* **38**, 325–350 (2017). 28651365 10.1210/er.2015-1114 PMC5546879

[50] Turner, C. H. Skeletal adaptation to mechanical loading. *Clin Rev Bone Miner Metab* **5**, 181–194 (2007).

[51] Xiao, Z. et al. Conditional deletion of Pkd1 in osteocytes disrupts skeletal mechanosensing in mice. *FASEB J* **25**, 2418–2432 (2011). 21454365 10.1096/fj.10-180299 PMC3219213

[52] Oest, M. E., Miller, M. A., Howard, K. I. & Mann, K. A. A novel in vitro loading system to produce supraphysiologic oscillatory fluid shear stress. *J Biomech* **47**, 518–525 (2014). 24275439 10.1016/j.jbiomech.2013.10.036 PMC3901248

[53] Boyle, W. J., Simonet, W. S. & Lacey, D. L. Osteoclast differentiation and activation. *Nature* **423**, 337–342 (2003). 12748652 10.1038/nature01658

[54] Gerbaix, M., Vico, L., Ferrari, S. L. & Bonnet, N. Periostin expression contributes to cortical bone loss during unloading. *Bone* **71**, 94–100 (2015). 25445447 10.1016/j.bone.2014.10.011

[55] Troy, K. L. et al. Bone adaptation in adult women is related to loading dose: a 12-month randomized controlled trial. *J Bone Miner Res* **35**, 1300–1312 (2020). 32154945 10.1002/jbmr.3999 PMC7363573

[56] Sasaki, T. et al. Spatiotemporal regulation of c-Fos by ERK5 and the E3 ubiquitin ligase UBR1, and its biological role. *Mol Cell* **24**, 63–75 (2006). 17018293 10.1016/j.molcel.2006.08.005

