# Supplementary material for: Exercise-stimulated primary cilia on preosteoclasts promote periosteal-bone formation
Source: Exp Mol Med. 2026 Jul 1;58(7):2168–83. doi: 10.1038/s12276-026-01765-5 (PMC13434781; doi:10.1038/s12276-026-01765-5)
Supplement: Supplementary file 1 [file 12276_2026_1765_MOESM1_ESM.pdf]

Supplementary Information

Exercise-stimulated primary cilia on preosteoclasts promote periosteal-bone formation

Jin-Man Kim[1], Young-Sun Lee[1], Min Ji Kim[1], Yewon Kim[1], Ho Kyoung Kim[1], Sung-Ah Moon[2], Hanjun Kim[1], Han Jin Cho[1], Sunghoon Kim[3], Seung Hun Lee[4], Min-Seon Kim[4], Young-Bum Kim[5] and Jung-Min Koh[4,*]

[1]Asan Institute for Life Sciences, Asan Medical Center, Seoul, 05505, Republic of Korea

[2]AMIST, Asan Medical Center, University of Ulsan College of Medicine, Seoul, 05505, Republic of Korea

[3]R&D Center, New Drug Research Institute, Hanlim Pharm. Co., Ltd, Seoul 06634, Republic of Korea

[4]Division of Endocrinology and Metabolism, Asan Medical Center, University of Ulsan College of Medicine, Seoul, 05505, Republic of Korea

[5]Division of Endocrinology, Diabetes, and Metabolism, Beth Israel Deaconess Medical Center and Harvard Medical School, Boston, MA, 02115, USA.

[*]Correspondence: Jung-Min Koh, E-mail: jmkoh@amc.seoul.kr

This file includes:

Supplementary Materials and Methods

Supplementary Table 1

Supplementary References

Supplementary Figures 1 to 10

Supplementary Materials and Methods

Antibodies, inhibitors, and constructs

We used antibodies against the following proteins: TGF-β1 (2 μg/mL, R&D Systems 9016), TGF-β2 (2 μg/mL, R&D Systems AB-112-NA), TGF-β3 (2 μg/mL, R&D Systems 20724), IGF-I (2 μg/mL, R&D Systems AF791), IGF-II (2 μg/mL, R&D Systems AF792), periostin (5 μg/mL, R&D Systems AF2955), integrin αvβ3 (10 μg/mL, Bioss BS-1310R), F4/80 (5 μg/mL, Invitrogen 14-4801-85), and Ctsk (10 μg/mL, Proteintech 11239-1-AP). The inhibitors used in this study are as follows: Odanacatib (100 nM), MMP9 Inhibitor I (10 nM, Millipore 444278), XAV939 (1 μM, Sigma-Aldrich X3004), L-NAME (1 mM, TOCRIS 0665), MG123 (200 nM, AG Scientific M1157), Bortezomib (100 nM, Selleck Chemicals S1013), BAPTA (10 μM, TOCRIS 2787), and Cyclopamine (1 μM, Sigma-Aldrich 239804).

To induce the expression of Dvl2 or mutant (Dvl2 S143A) in HEK293T cells, the 3× Flag Dvl2 wt was obtained from Addgene (Plasmid #24802) and the Dvl2 S143A mutant was generated with a QuickChange® II site-directed mutagenesis kit (Agilent Technologies). The Flag-tagged periostin plasmid was obtained from GenScript (0Hu16448D). The following mammalian expression constructs were purchased from Sino biotechnology: HA-c-Fos (HG10075-NY), His-ERK5 (HG10024-CH), and Myc-Ubiquitin B (RG80692-CM).

Genotyping

For genotyping analysis of the mice, the following primers were used: *Ctsk-cre* sense, 5′- AAT GCT TCT GTC CGT TTG C-3′ and antisense, 5′-CGG CAA CAC CAT TTT TTC TG-3′; *Col2.3-cre* sense, 5′-CTC TGG CTG TGG CCA CAG CTG AC-3′ and antisense, 5′-CC GGT TAT TCA ACT TGC ACC ATG C-3′; *Cx3cr1-cre* sense, 5′-GCT CTA CTT CAT CGC ATT CC-3′ and antisense, 5′-GCC TTA AGC AGT GAC TGA C-3′; Ift88^{loxP} sense, 5′-GAC CAC CTT TTT AGC CTC CTG-3′ and antisense, 5′-AGG GAA GGG ACT TAG GAA TGA-3′; and Kif3a^{loxP} sense, 5′-AGG GCA GAC GGA AGG GTG G-3′ and antisense, 5′-TCT GTG AGT TTG TGA CCA GCC-3′.

Cell culture and preparation of BMMϕ, preosteoclasts, and osteoclasts

HEK293T cells and MC3T3-E1 cells were obtained from American Type Culture Collection and maintained

respectively in Dulbecco's modified Eagle's medium (DMEM) and α-minimum essential medium (α-MEM) containing 10% fetal-bovine serum (FBS). To prepare BMMϕ, preosteoclasts, and osteoclasts, BMCs were harvested from the femurs and tibia of mice. The cells were centrifuged, treated with red-blood-cell lysis buffer (Sigma-Aldrich), and incubated overnight in α-MEM containing 10% FBS. To generate BMMϕ, non-adherent cells were collected and cultured with α-MEM containing 10% FBS and 30 ng/mL M-CSF. BMMϕ were seeded into 6-well culture plates and differentiated into preosteoclasts with 30 ng/mL M-CSF and 100 ng/mL RANKL for 2 days. At this timepoint, preosteoclasts predominated: TRAP+ mononuclear cells constituted >95% of all cells. To generate osteoclasts, the preosteoclasts were incubated with M-CSF and RANKL for another 2 days. TRAP+ multinucleated cells (>3 nuclei) were considered osteoclasts.

Osteoblast differentiation

To generate osteoblasts, calvarial-osteoblast precursors were harvested from the calvaria of newborn mice by sequential digestion with collagenase (Gibco 17018029) and trypsin. The harvested cells (4×10^4 cells) were seeded into 12-well culture plates and cultured in α-MEM containing 10% FBS, 10 mM β-glycerophosphate (Sigma-Aldrich), and 50 μg/mL ascorbic acid (Sigma-Aldrich) in the presence of 1 μg/mL recombinant periostin proteins (R&D Systems 3548-F2-050) for 3–4 days.

Osteoblast ALP activity was determined by washing the cells with PBS, resuspending them with buffer containing 50 mM Tris-HCl (pH 7.4) and 1 mM PMSF, sonicating and centrifuging the cells, and then treating the supernatants with alkaline buffer (Sigma-Aldrich) containing 1 mM $MgCl_2$ and 50 mM 4-nitrophenyl phosphate (Sigma-Aldrich) for 15 min at 37°C. Stop solution (0.3 N NaOH) was then added to the samples and the OD at 405 nm was measured with a microplate reader (Tecan Group Inc.). ALP activity was normalized to total protein content in each sample.

To determine osteoblast formation of bone nodules, calvarial-osteoblast precursors, periosteum-derived progenitor cells, or MC3T3-E1 cells were differentiated into osteoblasts with α-MEM containing 10% FBS, 10 mM β-glycerophosphate, and 50 μg/mL ascorbic acid in the presence of 1 μg/mL recombinant periostin proteins for 14 or 21 days. The cells were fixed with 70% ethanol and stained with 40 mM Alizarin Red S solution. The samples were washed with distilled water and dissolved with 10% cetylpyridinium chloride. The eluted samples

were quantified by measuring the OD at 570 nm with a microplate reader (Tecan Group Inc.).

RNA interference and qRT-PCR

DNA oligonucleotides encoding shRNAs specific for the coding regions of *Ift88* (5′-GCT CAA GTT CTT TGC CAG ATA-5′) and *Kif3a* (5′-TCC GCC AGT TTC AGA AAG AAA-3′) were subcloned into pLKO.1 lentiviral expression vector (Addgene) (https://www.addgene.org/tools/protocols/plko/). To generate lentiviral particles, HEK293T cells were co-transfected with plasmids encoding VSV-G or NL-BH, or the shRNAs using a Lipofectamine 3000 reagent (Invitrogen).

Total RNA was isolated from cells using a TRIzol reagent (Invitrogen). The RNA was reverse-transcribed into cDNA using a SuperScript^{TM} III reverse-transcriptase kit (Invitrogen) according to the manufacturer's protocol. Real-time PCR was performed using Lightcycler 480 SYBR green I mater (Roche). The primers are listed in Supplementary Table 1.

Primary-cilia staining

To detect primary cilia on femurs, paraffinized femur sections were deparaffinized, rehydrated, subjected to antigen retrieval (0.01 M citrate buffer, pH 6.0), washed with PBS, treated with 10 μg/mL proteinase-K in TE buffer (50 mM Tris-HCl, pH 8.0, 1mM EDTA, 0.5% Triton-X 100) for 10 min at room temperature (RT), washed with PBS containing 0.05% Triton X-100, blocked with 100 μL PBS containing 3% bovine-serum albumin (BSA) with/without 1.5 μg mouse IgG (for TRAP antibody) for 1 h at RT, and incubated at 4°C overnight with acetylated α-tubulin (1/500 dilution, Abcam ab179484), F4/80 (1/50 dilution, Abcam ab6640), and TRAP (1/100 dilution, Abcam ab212723) antibodies. After washing, the sections were incubated with Alexa Fluor 647- (1/250 dilution, Thermo Fisher Scientific A21244), 594- (1/250 dilution, Thermo Fisher Scientific A21209), and 555- (1/1000 dilution, Cell Signaling 4409) conjugated secondary antibodies.

To detect primary cilia on cells *in vitro*, bone-marrow cells (BMCs) harvested from mice were differentiated into preosteoclasts or osteoblasts by treatment with M-CSF/RANKL (preosteoclasts) or β-glycerophosphate/ascorbic acid (osteoblasts) for 3 or 21 days, respectively. Human peripheral blood monocytes (Lonza, CC-2702) were treated with M-CSF to generate macrophages for 6 days, while osteoclast differentiation was induced by stimulation with M-CSF and RANKL for 14 days.

The differentiated cells were fixed with 4% paraformaldehyde for 10 min, blocked with 3% BSA in PBS for 1 h, incubated with acetylated α-tubulin (1/500 dilution, Abcam ab179484) antibody at 4°C for 16 h, and then incubated with Alexa Fluor 555-conjugated antibody (1/1000 dilution, Cell signaling 4413) for 1 h. After 3 washes, nuclei were counterstained with DAPI. Primary cilia imaging was conducted with a confocal LSM710 microscope (Carl Zeiss).

Measurement of ciliated-cell number and primary-cilia length

Fluorescence images of acetylated α-tubulin (primary cilia) acquired from cells and femurs with the confocal LSM710 microscope (×400 magnification; Carl Zeiss) were used to create maximum projections of confocal z-stacks. The primary-cilia length was measured by using the ZEISS ZEN Blue software (version 3.8; Carl Zeiss). The number of ciliated cells in the differentiated osteoblast and preosteoclast samples was determined by counting the DAPI- and acetylated α-tubulin-positive cilia in 5 fields of view/sample. The primary-cilia lengths of >120 primary cilia/sample were also measured. The frequency of ciliated preosteoclasts in the femurs was determined in 189 and 169 TRAP+ mononucleated cells at the periosteal surfaces of femurs from 15 Ift88$^{loxP/loxP}$ mice and 12 Cx3cr1;Ift88$^{loxP/loxP}$ mice, respectively. The lengths of 96 and 76 primary cilia in the TRAP+ mononucleated cells at periosteal surfaces of femurs from 15 Ift88$^{loxP/loxP}$ mice and 12 Cx3cr1;Ift88$^{loxP/loxP}$ mice, respectively were also measured.

OSS and collection of preosteoclast CM

Preosteoclasts (4×10^5 cells) were seeded into 6-well culture plates and exposed to 310-rpm OSS for 0.5 to 24 h by using an orbital shaker (Mxi4t, FINEPCR) as previously described[1,2]. Maximal OSS (τ_{max}) was calculated as $\tau_{max} = a\sqrt{\eta\rho(2\pi f)^3}$, where a is the orbital radius of the rotation of the orbital shaker (0.95 cm), η is the culture-medium viscosity (0.0101 poise), ρ is the culture-medium density (0.9973 g/mL), and f is the frequency of rotation (rotation/s). The 310-rpm rotation frequency of the orbital shaker can induce approximately 17.6 dyne/cm^2 (1.76 Pa) shear stress. This shear-stress strength falls in the reported 1.67–24.55 Pa range of marrow shear stress that was imposed during cyclic loading in a previous study [3].

To collect conditioned media (CM), preosteoclast CMs were collected after 24 h with/without OSS exposure. After centrifugation, the CMs were filtered with a 0.22-μm filter and stored at -80°C until use.

Micro-computed tomography, histomorphometry, and immunofluorescence staining

Femurs were fixed with 4% paraformaldehyde for 16 h and scanned with a high-resolution micro-computed tomography scanner (Skyscan 1172 system). For histomorphometric analysis, the femurs were dissected, fixed, decalcified, and sectioned with a cryostat. Bone sections (5 μm) were deparaffinized, rehydrated, and subjected to TRAP or H&E staining. TRAP+ mononucleated cells at periosteal surfaces of femurs were identified as cited above [4].

For immunofluorescence staining, bone samples were stained with anti-F4/80 (1/50 dilution, Invitrogen 14-4801-85), anti-osteocalcin (1/200 dilution, Takara M173), anti-endomucin (1/25 dilution, Santa Cruz Biotechnology sc-65495), anti-Ctsk (1/50 dilution, Proteintech 11239-1-AP), TRAP (1/100 dilution, Abcam ab212723), or anti-periostin (1/25 dilution, R&D Systems AF2955) antibodies at 4°C for 16 h. The bone sections were washed and incubated with Alexa Fluor 594- (1/250 dilution, Thermo Fisher Scientific A21209 or A11058), 555- (1/250 dilution, Cell Signaling 4409), and 647- (1/250 dilution, Thermo Fisher Scientific A21244) secondary antibodies for 1 h at RT. The sections were counterstained with DAPI and observed with a confocal LSM710 microscope (Carl Zeiss). TRAP+ mononucleated cells in femurs were counted as described previously [5].

Bone-formation rate and serum ELISA assays for CTX, P1NP, and periostin

Mice were injected with calcein (30 mg/kg of body weight) at 10 and 3 days (Ex) or 14 and 3 days (Un) before sacrifice. Femur sections were analyzed using a semiautomatic image-analyzing system (Histometry RT Digitizer; System Supply Ltd) and the BIOQUANT program (Bio-Quant, Inc.). Serum CTX, P1NP, and periostin were measured using CTX ELISA kit (IDS Ltd., AC-02F1), P1NP ELISA kit (IDS Ltd., AC-33F1), and periostin ELISA kit (AdipoGen, AG-45B-0005-Kl01), respectively. Cleaved periostin in serum was also measured as described previously [6].

Periostin cleavage assay

Recombinant human His-tagged periostin proteins (1 μg, R&D Systems, 3548-F2-050) or human His-tagged periostin isoform 3 (1 μg, Sino Biological 10299-H08H for inhibition assay) were incubated with recombinant Ctsk (0.1 μg, Sigma-Aldrich 219461) in sodium-acetate buffer (pH 5.0 or pH 7.0) at 25°C for 2 h. To inhibit Ctsk,

100 nM Odanacatib or 10 μg/mL Ctsk-blocking antibody (ProteinTech, 11239-1-AP), which were supplied in sodium-acetate buffer (pH 7.0), were added to the incubated samples at 25°C. After 2 h incubation, 2× SDS sample buffer was added to the digested periostin samples and the samples were subjected to Coomassie brilliant-blue staining or western blotting.

Migration assay

Calvarial osteoblast precursors or periosteum-derived progenitor cells (1×10^5 cells) were seeded onto the upper chamber of a transwell plate in serum-free α-minimum essential medium (α-MEM). α-MEM supplemented with 10% (calvarial osteoblast precursor assay) or 50% (periosteum-derived progenitor cell assay) CMs from preosteoclasts was added to the lower chamber. The calvarial osteoblast precursors and periosteum-derived progenitor cells were allowed to migrate at 37°C for 5 h and 7 h, respectively.

For the inhibition migration assay, 50% CMs from preosteoclasts were pre-incubated with recombinant human periostin proteins (0.6 μg, R&D Systems, 3548-F2-050) in the presence or absence of cathepsin enzymes, including cathepsin K (Ctsk; Sigma-Aldrich, 219461), cathepsin B (Ctsb; R&D Systems, 953-CY), cathepsin L (Ctsl; R&D Systems, 952-CY), or cathepsin S (Ctss; R&D Systems, 1183-CY) (10 ng each), with or without HL235 (1 μM), for 1 h at 37°C. The pretreated conditioned media were then added to the lower chamber of a transwell plate. Periosteum-derived progenitor cells were seeded onto the upper chamber in serum-free α-MEM and allowed to migrate for 6 h at 37°C. The migrated cells were fixed with 4% formaldehyde for 15 min, stained with 1% crystal violet for 1 h, photographed, and counted. In parallel, the amount of recombinant human periostin remaining in the lower chamber was quantified using a human periostin ELISA kit (AdipoGen, AG-45B-0004-Kl01) according to the manufacturer’s instructions.

Cathepsin isoforms activity assay

Activities of cathepsin isoforms were measured using specific fluorometric assay kits (Abcam; ab65300, ab65303, ab65306, and ab65307) according to the manufacturer’s instructions. Briefly, cells were lysed in the provided lysis buffer (Abcam) on ice for 10 min and centrifuged at 12,000 × g for 10 min at 4°C to remove debris. The supernatants were collected and incubated with the corresponding fluorogenic substrates supplied in each kit. The reaction mixtures were incubated for 1 h at 37°C, and fluorescence intensity was measured using a microplate

fluorometer (Tecan Infinite M200PRO) at the recommended excitation and emission wavelengths. For inhibition assays, recombinant human cathepsin enzymes were incubated with their corresponding fluorogenic substrates in reaction buffer [50 mM 2-(N-morpholino)ethanesulfonic acid (MES), pH 5.5, 2 mM DTT, 2.5 mM EDTA, 10% DMSO, and 0.01% Triton X-100] in the presence of various concentrations of odanacatib or HL235. The reaction mixtures were pre-incubated for 30 min at 37°C before substrate addition, and fluorescence signals were measured using a microplate reader (Tecan Infinite M200PRO). Enzyme activities were calculated according to the manufacturer's protocols and normalized to control samples. Dose–response curves were fitted using nonlinear regression with a variable-slope three-parameter model in GraphPad Prism (version 8.3.0; Graphpad Software), and IC_{50} values were derived from the fitted curves.

mRNA-sequencing data, mRNA expression, and GSEA

The analysis pipeline focused on processing RNA-Seq data from *Mus musculus* (*mm10*). The process commenced with Tophat v2.0.13, incorporating the Bowtie v2.2.3 algorithm for read alignment [7,8]. The reference genome and annotation data were sourced from the UCSC table browser (http://genome.uscs.edu). Tophat utilized gene-annotation information during execution. Cufflinks v2.2.1 was employed for transcript assembly and abundance estimation, incorporating the '--max-bundle-frags 50000000' option to correct sequence-expression count bias. Default options were used for other parameters. For variant calling, STAR aligner [9] trimmed reads to *Mus musculus* (*mm10*), and SAMTOOLS and BCFTOOLS handled variant calling with filtering based on RMS mapping quality ($Q \geq 20$) and read depth ($d \geq 100$) in the varFilter module of vcftools. Moreover, Defuse 0.8.1 was applied to detect novel or known fusion genes in the RNA-Seq data [10]. To analyze mRNA expression, the initial raw data, measured in FPKM (Fragments Per Kilobase of exon model per Million mapped fragments) for each gene in every sample, was processed using the Cufflinks software. Genes with zeroed FPKM values in more than one sample were excluded. To facilitate $\log_{10}$ transformation, 1 was added to the FPKM value of the filtered genes. The filtered data underwent logarithmic transformation and were normalized using the quantile-normalization method. Our final determination of significance was based on adjusting for |fold change|≥1.5 and raw P-value <0.05. To gain insights into the biological significance of the large list of differentially-expressed genes (DEGs), we conducted gene-functional annotation analysis by using GSEA, which is accessible at http://www.broadinstitute.org/gsea/index.jsp.

Subcellular fractionation

Nuclear and cytosolic fractions were prepared by using NE-PER nuclear cytoplasmic extraction reagents (Pierce) according to the manufacturer's instructions. The cell pellet was vortexed and incubated on ice for 10 min. The sample was then subjected to ice-cold CER II, vortexed, and incubated on ice for 1 min. After centrifuging the sample, the supernatant was collected as the cytoplasmic fraction. The pellet was suspended in ice-cold NER and incubated on ice for 40 min with vortexing for 15 s every 10 min. After centrifugation, the supernatant was harvested as the nuclear fraction. The cytoplasmic and nuclear fractions were analyzed by western blotting.

Transfection and immunoprecipitation

HEK293T cells were transfected with His-tagged ERK5 construct and then treated with 200 μg/mL hygromycin B (Thermo Fisher Scientific) to select ERK5-expressing stable cells. The generated stable cells were co-transfected with Flag-Dvl2 wt or mt (S143A), HA-c-fos, and myc-Ubiquitin. After 24 h, the cells were treated with MG132 (200 nM) for 16 h and then lysed in lysis buffer (50 mM Tris-HCl, pH 7.4, 150 mM NaCl, 1 mM EDTA, and 1% Triton X-100) supplemented with protease inhibitor cocktail (Sigma-Aldrich). The cell lysates were pre-cleared with A/G agarose beads (Santa Cruz Biotechnology) for 1 h at 4°C and incubated with primary antibody and A/G agarose beads overnight at 4°C. After centrifugation, the immunoprecipitates were washed with lysis buffer 5 times and 2× SDS sample buffer was added. The denatured samples were analyzed with western blotting.

Immunofluorescence staining of Dvl2

Bone marrow macrophages (4×10^5 cells) were seeded in a μ-Dish 35-mm (Ibidi) in α-MEM culture medium containing M-CSF/RANKL and then cultured for 48 h. The cells were exposed to OSS at 37°C for 30 min or 60 min, fixed with 4% paraformaldehyde for 15 min, and permeabilized with 10 mM sodium-citrate buffer containing 0.1% Triton X-100 for 10 min. After washing with PBS, the cells were blocked with 2% BSA for 1 h and then incubated with anti-phosphorylated Dvl2 (pDvl2, 1/250 dilution, Abcam ab124933) or anti-total Dvl2 (tDvl2, 1/250 dilution, Cell signaling 3216) at 4°C overnight. After washing, the samples were incubated with an Alexa Fluor 555 secondary antibody (1/250 dilution, Cell signaling 4413) for 1 h at room temperature (RT) and then

counterstained with DAPI. All confocal images were obtained with a confocal LSM710 microscope (Carl Zeiss). The fluorescence intensity of pDvl2 or tDvl2 was analyzed by using ZEISS ZEN software (version 3.8; Carl Zeiss). The mean fluorescence intensity (MFI) was determined by dividing the total fluorescence intensity by the number of cells.

Cleaved periostin ELISA assay

A plate (Thermo Fisher Scientific 468667) was coated with a rabbit polyclonal antibody raised against periostin (amino acids 97-230, 5 μg/ml, Abbexa abx100627) overnight at 4°C. After rinsing with PBS, the wells were blocked with 5% BSA for 1 h at RT. Recombinant periostin FAS1 protein (amino acids 88-245, as a control for quantification) or mouse serum samples (1/50 dilution) were incubated for 2 h at RT. After washing, a biotinylated rabbit polyclonal periostin antibody (amino acids 97-230, 1/1000 dilution, USBiological Life Sciences 549208) was added to the wells. After incubation for 1 h at RT, the wells were washed and horseradish peroxidase-labeled streptavidin was added to each well. After 30-min incubation, the wells were washed and the substrate TMB solution was added for 10–15 min in the dark. The reaction was stopped by adding sulfuric-acid solution. Absorbance at 450 nm was read with a microplate reader (Tecan infinite M200PRO).

Preparation of periosteum-derived progenitor cells, flow-cytometry evaluation, and cell sorting

Periosteum-derived progenitor cells were harvested from mice as previously described [11]. Briefly, 6 week-old male C57BL/6 mice were sacrificed and their tibias and femurs were dissected. After cutting the epiphyses, the bones were flushed to remove all bone-marrow cells. The bones were cut into smaller sizes and cultured in α-MEM containing 10% FBS. The cells migrated out of the explanted tissues within 3 days. After 7 days, the bones were removed, the cells were washed with PBS, and culture medium was added. After 14 days of culture, the cells were trypsinized and subcultured for up to two passages. When the desired cell numbers were attained, the cells were used for flow-cytometric and cell-sorting experiments. For flow-cytometric evaluation, the cells were stained with CD11b-PE (Invitrogen 12-0112-82), CD31-PE (Invitrogen 12-0311-82), CD45-PE (Invitrogen 12-0451-82), CD34-PE (Invitrogen RM3604), CD29-PE (Invitrogen 12-0291-82), CD105-PE (Invitrogen 12-1051-82), or Sca1-APC (Invitrogen 17-5981-82) antibodies. These antibodies were used to characterize the periosteum-derived progenitor cells as described previously [11,12]. Flow cytometry was performed with a BD FACS Canto II flow

cytometer (BD Biosciences). For cell sorting, the antibody-labeled cells were resuspended in PBS containing 1% FBS and 2 mM EDTA and then sorted with a BD FACS Aria II cell sorter (BD Biosciences).

***In vitro* adipogenic and chondrogenic differentiation assays of periosteum-derived progenitor cells**

To induce adipogenic differentiation, the cells (1×10^5) were seeded into a 24-well culture plate for 24 h. The medium was replaced with adipogenic differentiation medium (Gibco, A10070-01) and the cells were cultured for 14 days with medium changes every 2–3 days. The cells were fixed with 4% paraformaldehyde and stained with Oil Red O solution (Sigma B8438) according to the manufacturer's protocol. To induce chondrogenic differentiation, 1×10^5 cells were plated in a 24-well plate. After 1 day, the cells were cultured with chondrogenic differentiation medium (Gibco, A10071-01). The medium was changed every 2–3 days for 14 days and the cells were stained with Alcian blue solution (Sigma O1391) according to the manufacturer's protocol.

Western blot

Cells were rinsed with PBS and lysed with RIPA buffer containing a mixture of protease inhibitors (Sigma-Aldrich), 1 mM β-glycerophosphate, and 1 mM Na_3VO_4. The proteins were separated by sodium dodecyl sulfate (SDS)-polyacrylamide gel electrophoresis and transferred onto a 0.2-μm nitrocellulose membrane. Primary antibodies were used to probe the membrane and appropriate secondary antibodies (Cell Signaling) were applied. Immunoreactive signals were visualized with enhanced chemiluminescence reagents (Dynebio ECL Dura or Pico plus). Band density was determined with an Image Gauge (version 4.0; Fujifilm).

Statistical analysis

All quantitative data are represented as mean±SD or SEM. GraphPad Prism was used for all analyses. When comparing two groups, unpaired two-tailed Student's t-test was used. When more than two groups were compared, one-way or two-way ANOVA was performed, followed by post-hoc testing with Tukey's or Sidak's multiple comparison test. *P*-values <0.05 were considered statistically significant.

Supplementary Table 1. Primer sequences for quantitative real-time PCR.

Primers	Sense (5'-3')	Antisense (5'-3')
Ift88	CCT TTG GAA GCC AAA AAT GA	TCT CCA CAA CTG TTG GCA AT
Kif3a	TGT GTA TAA CTT AAC TGC AAG ACC AA	TTG CCT GTC CCA GTC TGT C
Pdgfa	AGTTTGCAAGACCAGGACGG	GCTTCACCTCCACACATGGG
Pdgfb	TGCTGAGCGACCACTCCATC	CTCGGGTCATGTTCAAGTCC
Tgfβ1	GCT CCC CTA TTT AAG AAC ACC C	TTG AGG TTG AGG GAG AAA GC
Tgfβ2	CTC TGG CTT TCA TTT GGC TTG	TCC TTG CAT TCA CAC CTC AG
Tgfβ3	AAG AGA AGC AAG GGA CAG AAG	GCT GGG ATG AGG GAT TAT GTA C
Igf1	TGG AGT GGG AAA AGC AAG AG	CGA GAG GTG GAG TGA TTT GAC
Igf2	CCT TTC ACG TTC ACT CTG TCT C	CTT TAG GGT ATC TTA GGG CTG G
Nos1	AAG TCC TAA ATC CAG CCG ATC	TCC AGC CGT TCA ATG AGT G
Nos2	GGC AGC CTG TGA GAC CTT TG	GCA TTG GAA GTG AAG CGT TTC
Nos3	TCC GGA AGG CGT TTG ATC	GCC AAA TGT GCT GGT CAC C
Ctsk	ATATGTGGGCCAGGATGAAAGTT	TCGTTCCCCACAGGAATCTCT
Mmp9	TGTCTGGAGATTCGACTTGAAGTC	TGAGTTCCAGGGCACACCA
Acp5	CGT CTC TGC ACA GAT TGC AT	AAG CGC AAA CGG TAG TAA GG
c-fos	TAA GTA GTG CAG CCC GGA GT	GTC CGG TTC CTT CTA TGC AG
Gapdh	CTCCACTCACGGCAAATTCA	GCCTCACCCCATTTGATGTT
18S	CTC AAC ACG GGA AAC CTC AC	CGC TCC ACC AAC TAA GAA CG

Supplementary References

1 Kim, H. *et al.* Different Effects of Orbital Shear Stress on Vascular Endothelial Cells: Comparison with the Results of In Vivo Study with Rats. *Vascular specialist international* 31, 33-40, doi:10.5758/vsi.2015.31.2.33 (2015).

2 Dardik, A. *et al.* Differential effects of orbital and laminar shear stress on endothelial cells. *Journal of vascular surgery* 41, 869-880, doi:10.1016/j.jvs.2005.01.020 (2005).

3 Metzger, T. A., Schwaner, S. A., LaNeve, A. J., Kreipke, T. C. & Niebur, G. L. Pressure and shear stress in trabecular bone marrow during whole bone loading. *Journal of biomechanics* 48, 3035-3043, doi:10.1016/j.jbiomech.2015.07.028 (2015).

4 Xie, H. *et al.* PDGF-BB secreted by preosteoclasts induces angiogenesis during coupling with osteogenesis. *Nature medicine* 20, 1270-1278, doi:10.1038/nm.3668 (2014).

5 Gao, B. *et al.* Macrophage-lineage TRAP+ cells recruit periosteum-derived cells for periosteal osteogenesis and regeneration. *The Journal of clinical investigation* 129, 2578-2594, doi:10.1172/JCI98857 (2019).

6 Rousseau, J. C., Sornay-Rendu, E., Bertholon, C., Chapurlat, R. & Garnero, P. Serum periostin is associated with fracture risk in postmenopausal women: a 7-year prospective analysis of the OFELY study. *The Journal of clinical endocrinology and metabolism* 99, 2533-2539, doi:10.1210/jc.2013-3893 (2014).

7 Trapnell, C., Pachter, L. & Salzberg, S. L. TopHat: discovering splice junctions with RNA-Seq. *Bioinformatics* 25, 1105-1111, doi:10.1093/bioinformatics/btp120 (2009).

8 Langmead, B., Trapnell, C., Pop, M. & Salzberg, S. L. Ultrafast and memory-efficient alignment of short DNA sequences to the human genome. *Genome biology* 10, R25, doi:10.1186/gb-2009-10-3-r25 (2009).

9 Dobin, A. *et al.* STAR: ultrafast universal RNA-seq aligner. *Bioinformatics* 29, 15-21, doi:10.1093/bioinformatics/bts635 (2013).

10 McPherson, A. *et al.* deFuse: an algorithm for gene fusion discovery in tumor RNA-Seq data. *PLoS computational biology* 7, e1001138, doi:10.1371/journal.pcbi.1001138 (2011).

11 Ude, C. C., Sharma, G. G., Shen, J. & O'Keefe, R. J. Isolation and Culture of Periosteum-Derived Progenitor Cells from Mice. *Methods in molecular biology* 2230, 397-413, doi:10.1007/978-1-0716-

1028-2_24 (2021).

12 Duchamp de Lageneste, O. *et al.* Periosteum contains skeletal stem cells with high bone regenerative potential controlled by Periostin. *Nature communications* 9, 773, doi:10.1038/s41467-018-03124-z (2018).

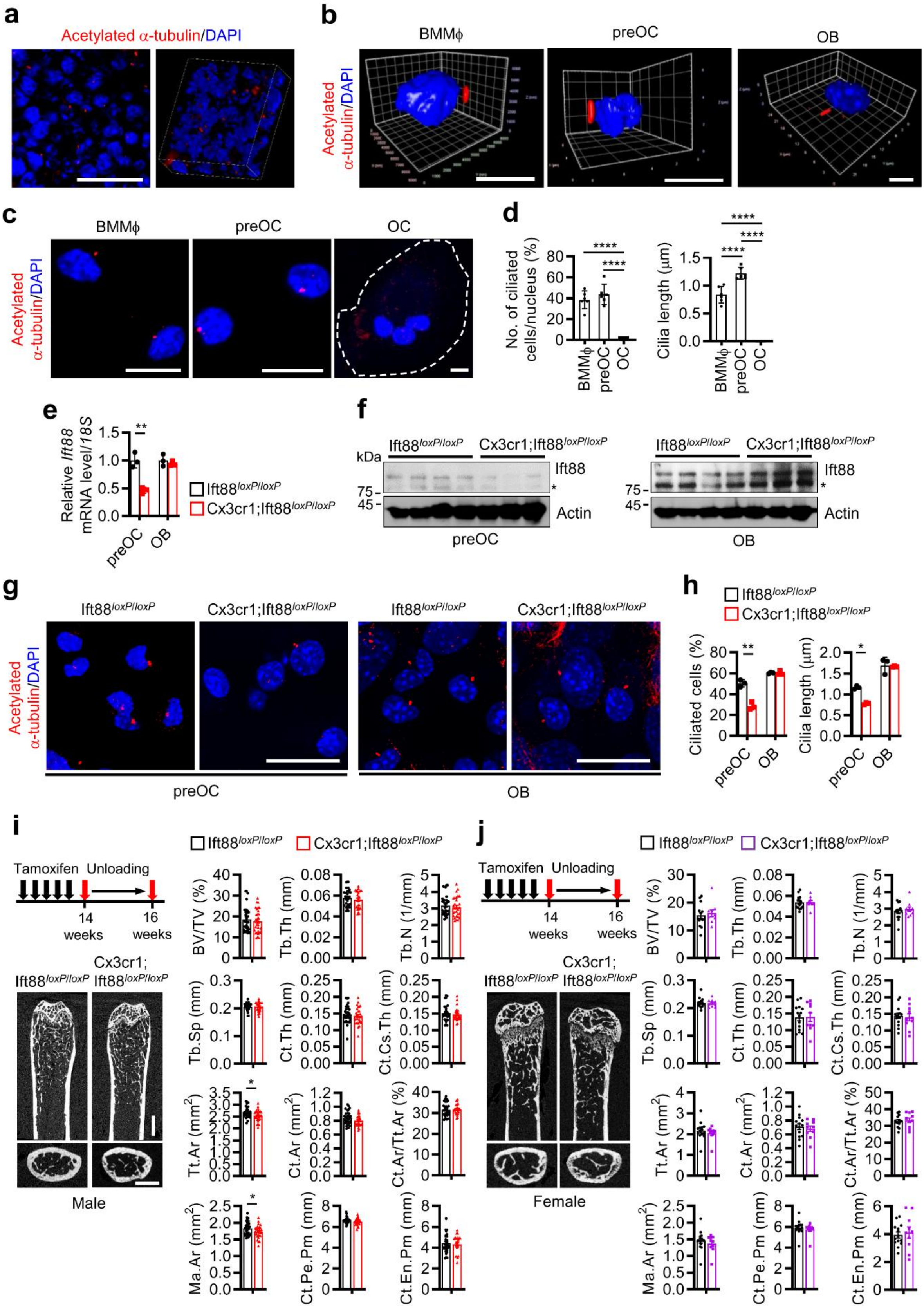

a
Acetylated α-tubulin/DAPI
b
BMMϕ
preOC
OB
Acetylated α-tubulin/DAPI
c
BMMϕ
preOC
OC
Acetylated α-tubulin/DAPI
d
No. of ciliated cells/nucleus (%)
Cilia length (μm)
BMMϕ
preOC
OC
e
Relative Ift88 mRNA level/18S
preOC
OB
Ift88loxP/loxP
Cx3cr1;Ift88loxP/loxP
f
Ift88loxP/loxP
Cx3cr1;Ift88loxP/loxP
kDa
Ift88
Actin
preOC
OB
g
Ift88loxP/loxP
Cx3cr1;Ift88loxP/loxP
Acetylated α-tubulin/DAPI
preOC
OB
h
Ciliated cells (%)
Cilia length (μm)
i
Tamoxifen
Unloading
14 weeks
16 weeks
Male
BV/TV (%)
Tb.Th (mm)
Tb.N (1/mm)
Tb.Sp (mm)
Ct.Th (mm)
Ct.Cs.Th (mm)
Tt.Ar (mm2)
Ct.Ar (mm2)
Ct.Ar/Tt.Ar (%)
Ma.Ar (mm2)
Ct.Pe.Pm (mm)
Ct.En.Pm (mm)
j
Female

Supplementary Fig. 1. Micro-CT of femurs of male and female Cx3cr1;Ift88$^{loxP/loxP}$ mice after hind-limb unloading. a Immunostaining of acetylated α-tubulin (red) in femurs of 16-week-old male C57BL/6 mice are shown in the 2D (left) and 3D (right) views. Scale bar, 20 μm. **b** 3D-reconstructed confocal images showing primary cilia (acetylated α-tubulin, red) in BMMϕ, preosteoclasts (preOC), and osteoblasts (OB). Scale bar, 5 μm. **c** and **d** Immunostaining of acetylated α-tubulin (red) in (**c**) human peripheral blood monocyte-derived BMMϕ, preOC, and osteoclasts (OC). Scale bar, 10 μm. (**d**) Frequency of ciliated cells and cilium lengths in BMMϕ (*n*=6), preOC (*n*=6), and OC (*n*=14). (**e**–**h**) Bone marrow cells of 16-week-old male Cx3cr1;Ift88$^{loxP/loxP}$ mice and their Ift88$^{loxP/loxP}$ littermates were differentiated into preosteoclasts (preOC) and osteoblasts (OB) with treating M-CSF/RANKL and ascorbic acid/β-glycerophosphate, respectively, as described in the Methods. (**e**) Real-time PCR (*n*=3) and (**h**) western blot for Ift88 were performed. Asterisks indicate non-specific bands. (**g**) Immunostaining of acetylated α-tubulin (red) images are shown. Scale bar, 20 μm. (**i**) The percentage of ciliated cells per nucleus (left) and cilia length (right) were measured (*n*=3). **i** and **j** Male (**i**) and female (**j**) Cx3cr1;Ift88$^{loxP/loxP}$ mice and their Ift88$^{loxP/loxP}$ littermates were suspended by their tails for two weeks. Representative micro-CT images of the proximal femurs of Cx3cr1;Ift88$^{loxP/loxP}$ mice at 16 weeks of age (*n*=30 **i**, *n*=10 **j**) and their Ift88$^{loxP/loxP}$ littermates (*n*=32 **i**, *n*=14 **j**) are shown in the left. Their quantitative results are shown in the right. BV/TV, trabecular bone volume per tissue volume; Tb.Th, trabecular thickness; Tb.N, trabecular number, Tb.Sp, trabecular spacing; Ct.Th, cortical thickness; Ct.Cs.Th, cortical cross-sectional thickness; Tt.Ar, total cross-sectional area inside the periosteal envelope; Ct.Ar, cortical area; Ma.Ar, marrow area; Ct.Pe.Pm, cortical periosteal perimeter; and Ct.En.Pm, cortical endosteal perimeter. Scale bar, 1 mm. Mean±SD (**d,e,h)** or mean±SEM (**i,j**) are shown. Statistical significance was determined by Student's unpaired two-tailed t-test (**i,j**) or one-way ANOVA followed by Tukey's test (**d**). Two-way ANOVA was performed followed by Sidak's post-hoc test (**e**,**h**). $*P < 0.05$, $**P < 0.01$, and $****P < 0.0001$.

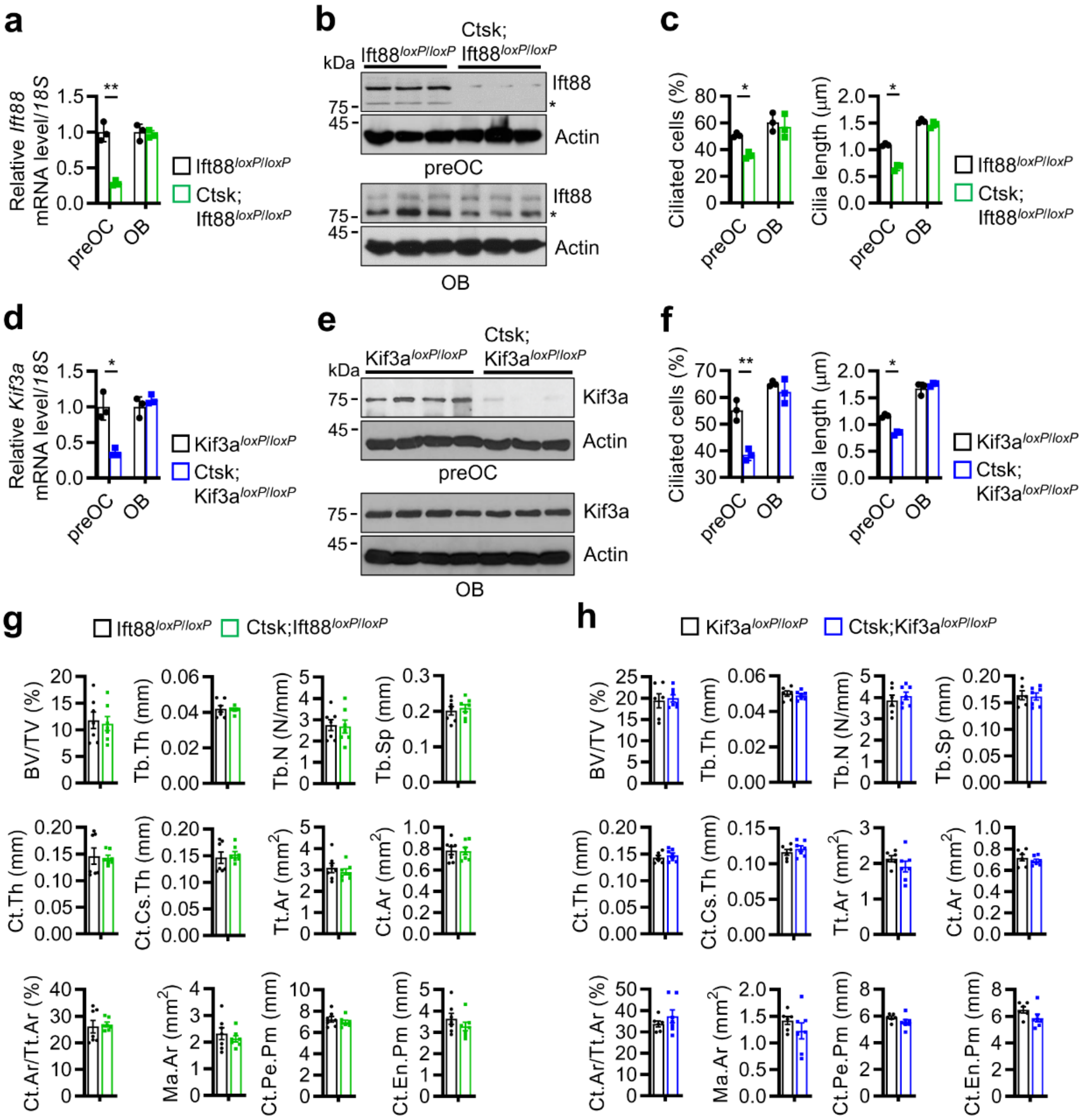

Supplementary Fig. 2. Micro-CT of femurs of male Ctsk;Ift88$^{loxP/loxP}$ and Ctsk;Kif3a$^{loxP/loxP}$ mice after hind-limb unloading. a–f Bone marrow cells of 16-week-old male (**a–c**) Ctsk;Ift88$^{loxP/loxP}$ and male (**d–f**) Ctsk;Kif3a$^{loxP/loxP}$ mice, and their respective Ift88$^{loxP/loxP}$ and Kif3a$^{loxP/loxP}$ littermates were differentiated into preosteoclasts (preOC) and osteoblasts (OB). (**a** and **d**) Real-time PCR (*n*=3) and (**b** and **e**) western blot for (**a** and **b**) Ift88 and (**d** and **e**) Kif3a were performed. Asterisks indicate non-specific bands. (**c** and **f**) The percentage of ciliated cells per nucleus (left) and cilia length (right) were measured (*n*=3). **g** and **h** Male (**g**) Ctsk;Ift88$^{loxP/loxP}$, male (**h**) Ctsk;Kif3a$^{loxP/loxP}$ mice, and their respective wild type littermates were suspended by their tails for two weeks. Quantitative micro-CT results of proximal femurs of (**g**) Ctsk;Ift88$^{loxP/loxP}$ (*n*=7) and Ift88$^{loxP/loxP}$ mice (*n*=7), and (**h**) Ctsk;Kif3a$^{loxP/loxP}$ (*n*=7) and Kif3a$^{loxP/loxP}$ mice (*n*=6) are shown. BV/TV, trabecular bone volume per tissue volume; Tb.Th, trabecular thickness; Tb.N, trabecular number, Tb.Sp, trabecular spacing; Ct.Th, cortical thickness; Ct.Cs.Th, cortical cross-sectional thickness; Tt.Ar, total cross-sectional area inside the periosteal envelope; Ct.Ar, cortical area; Ma.Ar, marrow area; Ct.Pe.Pm, cortical periosteal perimeter; and Ct.En.Pm, cortical endosteal perimeter. Mean±SD (**a–f**) or mean±SEM (**g**,**h**) are shown. Statistical significance was determined by Student's unpaired two-tailed t-test (**g**,**h**). Two-way ANOVA was performed followed by Sidak's post-hoc test (**a–f**). $^*P < 0.05$ and $^{**}P < 0.01$.

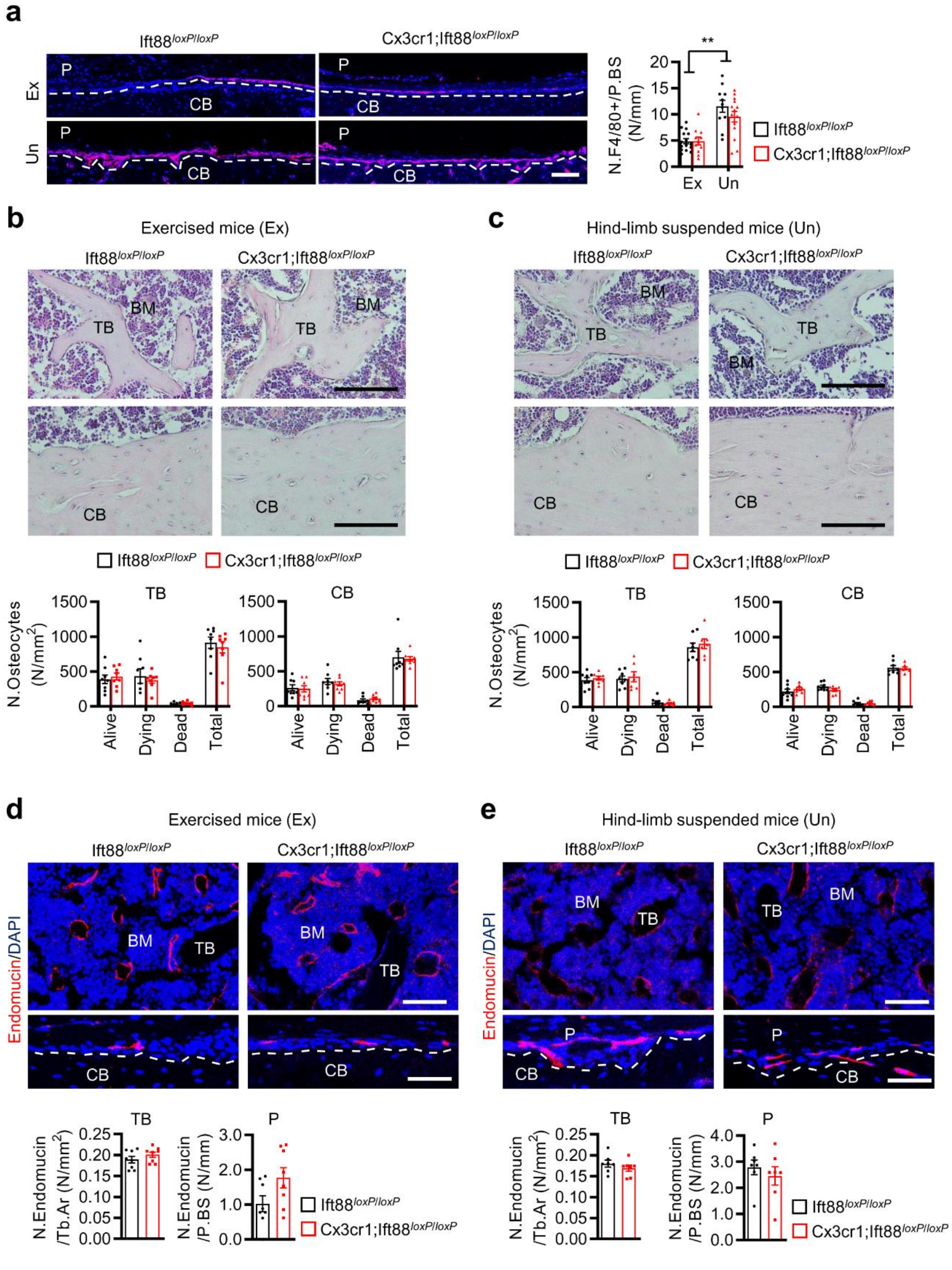
a
Ift88loxP/loxP
Cx3cr1;Ift88loxP/loxP
Ex
Un
P
CB
N.F4/80+/P.BS (N/mm)
**
Ex Un
b
Exercised mice (Ex)
TB
BM
c
Hind-limb suspended mice (Un)
N.Osteocytes (N/mm2)
Alive
Dying
Dead
Total
d
Endomucin/DAPI
N.Endomucin /Tb.Ar (N/mm2)
N.Endomucin /P.BS (N/mm)
e

Supplementary Fig. 3. Myeloid-specific deletion of Ift88 did not alter macrophage, osteocyte, or endomucin+ endothelial cell numbers. Mice underwent exercise (Ex) or were suspended by their tails (unloaded, Un), as described in **Fig. 1g** and **Supplementary Fig. 1f**. **a** Representative F4/80 (red) immunostaining images of femoral periosteal surfaces in Cx3cr1;Ift88$^{loxP/loxP}$ mice (n=11 Ex, n=14 Un) and Ift88$^{loxP/loxP}$ mice (n=15 Ex, n=11 Un). Scale bar, 100 μm. Right: F4/80+ macrophage number per periosteal-bone surface (N.F4/80+/P.BS). **b** and **c** Representative hematoxylin and eosin staining images of femurs of male Cx3cr1;Ift88$^{loxP/loxP}$ mice (n=8 Ex, n=7 Un) and their Ift88$^{loxP/loxP}$ littermates (n=8 Ex, n=8 Un) (**b**) under-exercising and (**c**) hind-limb suspension are shown in the upper part. Osteocyte numbers (N. Osteocytes) per trabecular (TB) and cortical (CB) bone areas were shown at the bottom of each. Scale bar, 100 μm. **d** and **e** Immunostaining images of endomucin (red) in femurs of male Cx3cr1;Ift88$^{loxP/loxP}$ mice (n=8 Ex, n=7 Un) and their Ift88$^{loxP/loxP}$ littermates (n=8 Ex, n=7 Un) (**d**) under-exercising and (**e**) hind-limb suspension are shown in the upper. Endomucin number per trabecular bone area (N.Endomucin/Tb.Ar) and periosteal bone surface (N. Endomicin/P.BS) were measured at the bottom of each. Scale bar, 50 μm. TB, trabecular bone; BM, bone marrow; CB; cortical bone; and P, periosteum. Mean±SEM are shown. Two-way ANOVA was performed followed by Sidak's post-hoc test (**a**). $^{**}P < 0.01$.

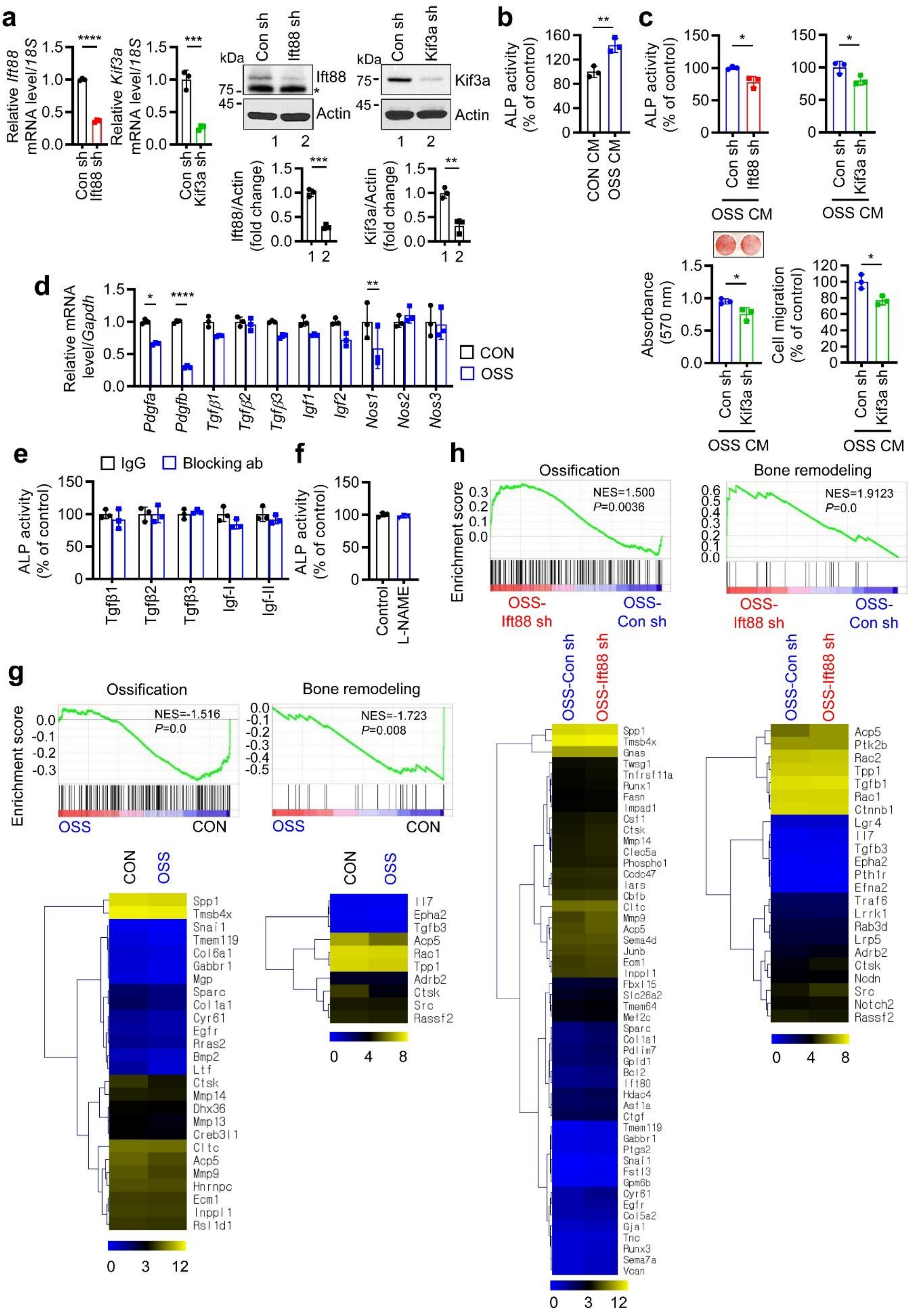
a
Relative Ift88 mRNA level/18S
Con sh
Ift88 sh
Relative Kif3a mRNA level/18S
Kif3a sh
kDa
Ift88
Actin
Kif3a
Ift88/Actin (fold change)
Kif3a/Actin (fold change)
b
ALP activity (% of control)
CON CM
OSS CM
c
OSS CM
Absorbance (570 nm)
Cell migration (% of control)
d
Relative mRNA level/Gapdh
Pdgfa
Pdgfb
Tgfβ1
Tgfβ2
Tgfβ3
Igf1
Igf2
Nos1
Nos2
Nos3
CON
OSS
e
IgG
Blocking ab
Tgfβ1
Tgfβ2
Tgfβ3
Igf-I
Igf-II
f
Control
L-NAME
g
Ossification
Bone remodeling
Enrichment score
NES=-1.516
P=0.0
NES=-1.723
P=0.008
OSS
CON
h
Ossification
Bone remodeling
NES=1.500
P=0.0036
NES=1.9123
P=0.0
OSS-Ift88 sh
OSS-Con sh

Supplementary Fig. 4. Shear stress-stimulated preosteoclast secretions, primary cilia-dependently, stimulated osteoblastic bone formation. a Knockdown efficiencies of *Ift88* and *Kif3a* in preosteoclasts was determined by real-time PCR (left) and western blot (right). Band densities are shown at the lower. The asterisk indicates non-specific bands. **b** Bone marrow macrophages were differentiated into preosteoclasts as described in the Methods and were exposed to orbital shear stress (OSS) for 24 h or not (CON). Calvaria osteoblast precursors were treated with preosteoclast conditioned media (CM), and analyzed by alkaline phosphatase (ALP) activity assay. **c** Preosteoclasts transfected with *Ift88* sh or *Kif3a* sh were exposed to OSS for 24 h and then collected CM. Calvaria osteoblast precursors were treated with the CMs, and their ALP activity assay, mineralization, and migration were measured as described in **Fig. 4a**. **d** Preosteoclasts were exposed with OSS for 24 h, and real-time PCR for the indicated genes was performed. **e** and **f** Calvaria osteoblast precursors were treated with preosteoclast CMs exposed to OSS for 24 h or not in the presence of (**e**) 2 μg/ml blocking antibodies against the indicated factor or control immunoglobulin G (IgG), and ALP activity was performed. Instead of blocking antibodies, (**f**) 1 mM L-N^G-nitro arginine methyl ester (L-NAME) was treated. **g** and **h** Preosteoclasts were exposed to (**g**) OSS for 24 h or not. Alternatively, preosteoclasts transfected with (**h**) *Ift88* sh or control sh (Con sh) were exposed to OSS. RNA-sequencing analysis was performed, and gene set enrichment analyses (GSEA) showed the enrichment of genes associated with ossification and bone remodeling. Heat map representation of the enriched genes based on their enrichment scores in GSEA profiles in OSS-stimulated (**g**) preosteoclasts and OSS-stimulated (**h**) *Ift88*–lacking preosteoclasts. Mean±SD (all, n=3) are shown. Statistical significance was determined by Student's unpaired two-tailed t-test (**a–c**). Two-way ANOVA was performed followed by Sidak's post-hoc test (**d**). $^*P < 0.05$, $^{**}P < 0.01$, $^{***}P < 0.001$, and $^{****}P < 0.0001$.

a OSS *vs* CON

Gene	Ossification		Bone remodeling	
	Rank	Rank metric score	Rank	Rank metric score
Ctsk	1	-1.160	1	-1.160
Acp5	2	-0.892	2	-0.892
Mmp9	3	-0.841	ND	NA
Ltf	4	-0.616	ND	NA
Col1a1	5	-0.433	ND	NA

b OSS-Ift88 sh *vs* OSS-Con sh

Gene	Ossification		Bone remodeling	
	Rank	Rank metric score	Rank	Rank metric score
Mmp9	1	1.086	ND	NA
Acp5	2	0.640	1	0.640
Ctsk	3	0.515	2	0.515
Csf1	4	0.416	ND	NA
Mmp14	5	0.414	ND	NA

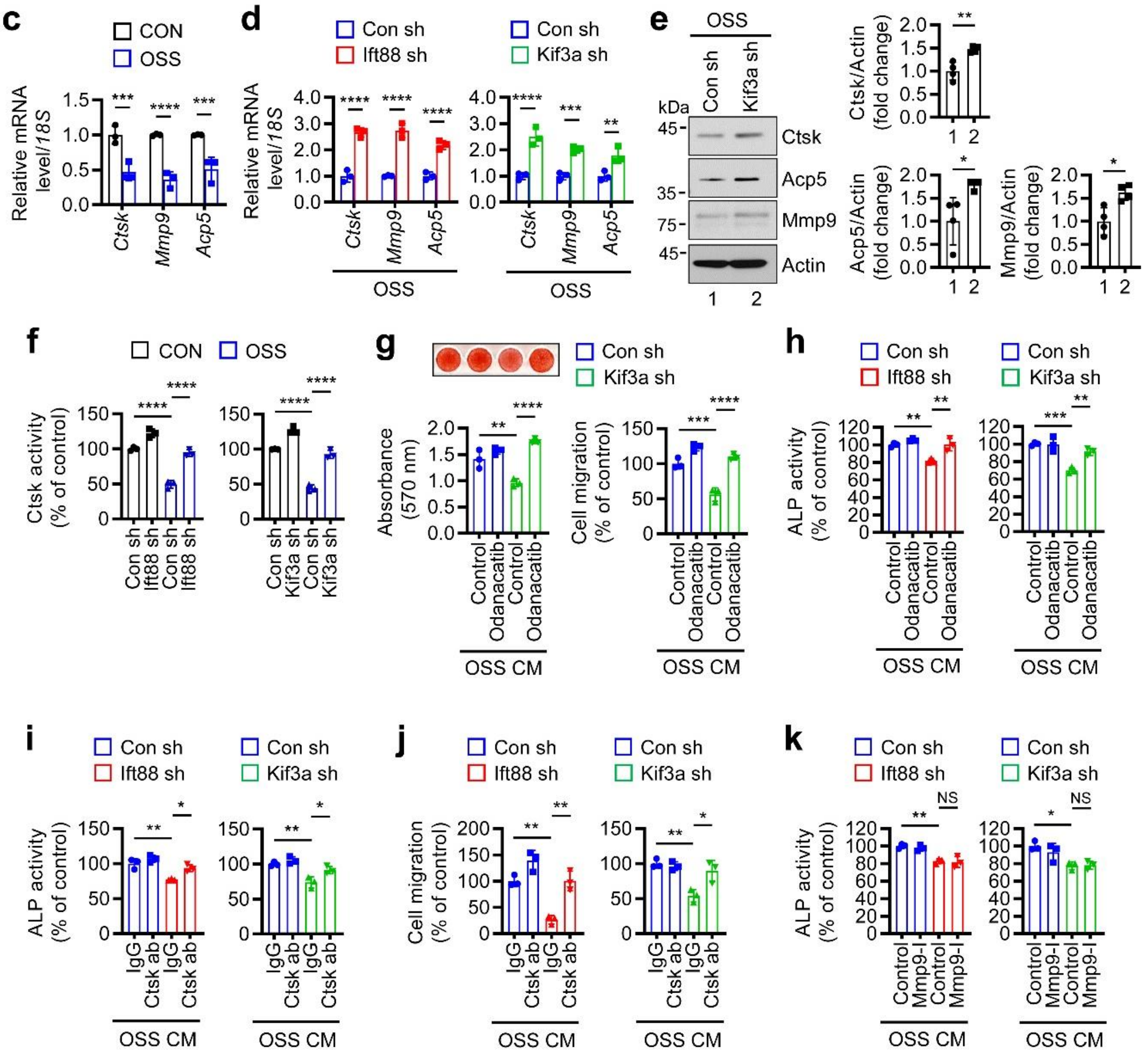

Supplementary Fig. 5. Suppressed cathepsin K production in preosteoclasts in response to shear stress, primary cilia-dependently, stimulated osteoblastic bone formation. a and **b** Top 5 enriched genes in gene set enrichment analyses of ossification and bone remodeling of **Supplementary Fig. 4g**, **h** are shown. Top 3 genes are marked in red. **c** and **d** Real-time PCR for cathepsin K (*Ctsk*), acid phosphatase 5 (*Acp5*), and matrix metallopeptidase-9 (*Mmp9*) were performed in preosteoclasts exposed to (**c**) OSS, and in those transfected with (**d**) *Ift88* sh or *Kif3a* sh. **e** Western blots for the indicated factors were performed in *Kif3a*-lacking preosteoclasts exposed to OSS. Band densities are shown in the right. **f** Preosteoclasts transfected with *Ift88* sh or *Kif3a* sh were exposed to OSS for 24 h, and Ctsk activity assays were performed. **g–k** Preosteoclasts transfected with *Kif3a* sh or *Ift88* sh were treated with (**g** and **h**) 100 nM odanacatib, (**i** and **j**) 10 µg/mL Ctsk blocking antibody, or (**k**) 10

nM MMP9 inhibitor I, exposed to OSS for 24 h, and their CM were harvested. Calvaria osteoblast precursors were treated with the CMs, and their (**g**) mineralization, (**g** and **j**) migration, and (**h**, **i**, **k**) ALP activity were measured. NS, not significant. Mean±SD [all, *n*=3 except **e** (*n*=4)] are shown. Statistical significance was determined by two-way ANOVA followed by Sidak's post-hoc test (**c**,**d**), student's unpaired two-tailed t-test (**e**), and one-way ANOVA followed by Turkey's test (**f–k**). *$P < 0.05$, **$P < 0.01$, ***$P < 0.001$, and ****$P < 0.0001$.

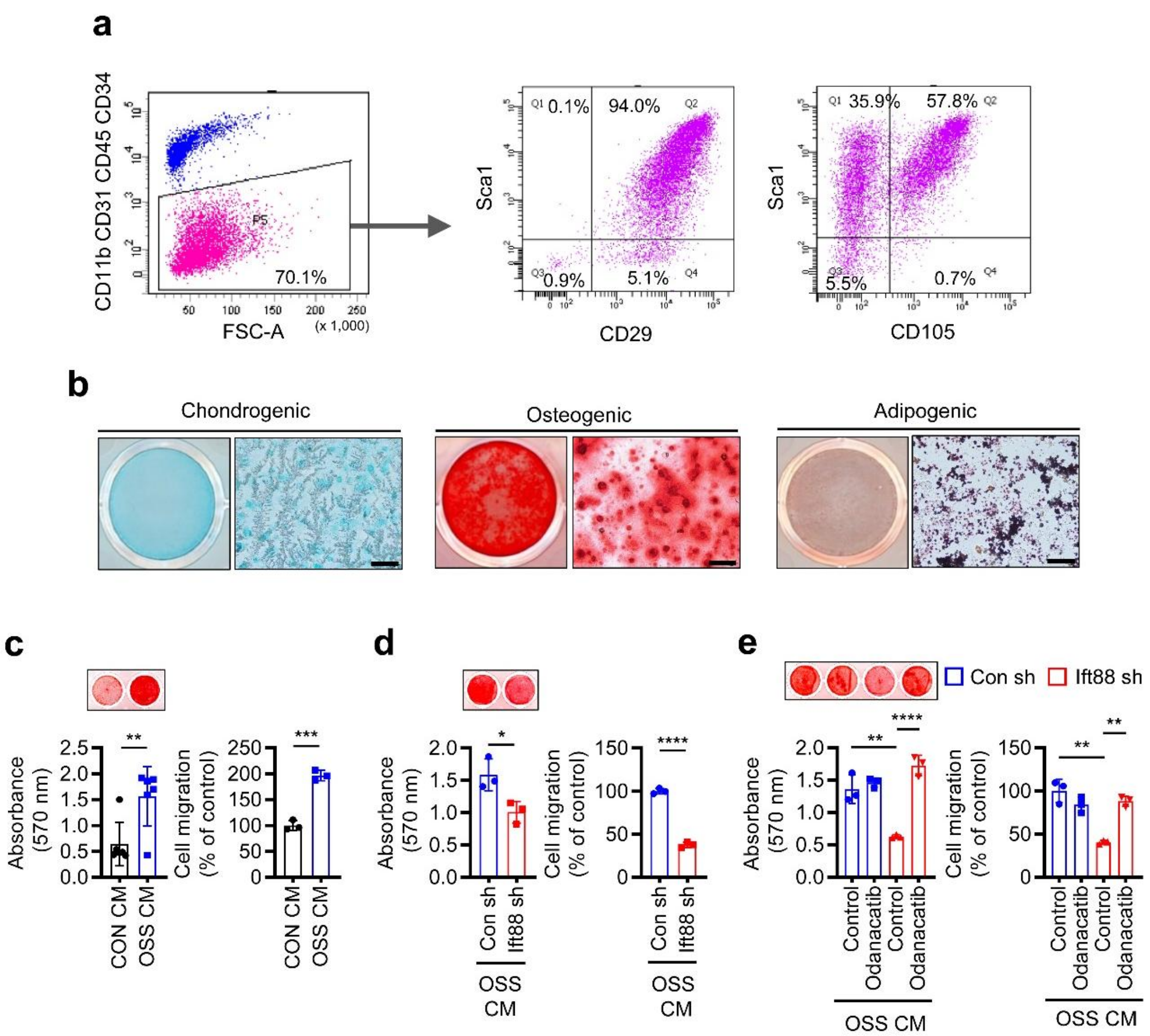

Supplementary Fig. 6. Collection and *in vitro* analyses of periosteum-derived progenitor cells. a Flow cytometry analyses of periosteum-derived progenitor cells harvested from male C75BL/6 mice. Periosteum-derived progenitor cells were negative for endothelial and hematopoietic markers (CD11b, CD31, CD45, and CD34) and positive for Sca1/CD29/CD105. **b** Sorted periosteum-derived progenitor cells were differentiated into chondrogenic, osteogenic, and adipogenic lineages and stained with Alcian Blue, Alizarin Red S, and Oil Red O, respectively. Scale bar, 100 µm. **c** Bone marrow macrophages were differentiated into preosteoclasts and were exposed to orbital shear stress (OSS) for 24 h or not (CON). Sorted periosteum-derived progenitor cells were treated with preosteoclast conditioned media (CM), and their mineralization (n=6) and migration (n=3) were measured as described in **Fig. 4a**. **d** Preosteoclasts transfected with *Ift88* sh or control sh (Con sh) were exposed to OSS for 24 h and then collected the OSS CM. Sorted periosteum-derived progenitor cells were treated with the CMs, and their mineralization (n=3) and migration (n=3) were performed. **e** *Ift88*-knockdown preosteoclasts were treated with 100 nM odanacatib, and exposed to OSS for 24 h. Sorted periosteum-derived progenitor cells were treated with the OSS CM, and their mineralization (n=3) and migration (n=3) were measured. Mean±SD are shown. Statistical significance was determined by Student's unpaired two-tailed t-test (**c**,**d**). One-way ANOVA was performed followed by Turkey's test (**e**). *$P < 0.05$, **$P < 0.01$, ***$P < 0.001$, and ****$P < 0.0001$.

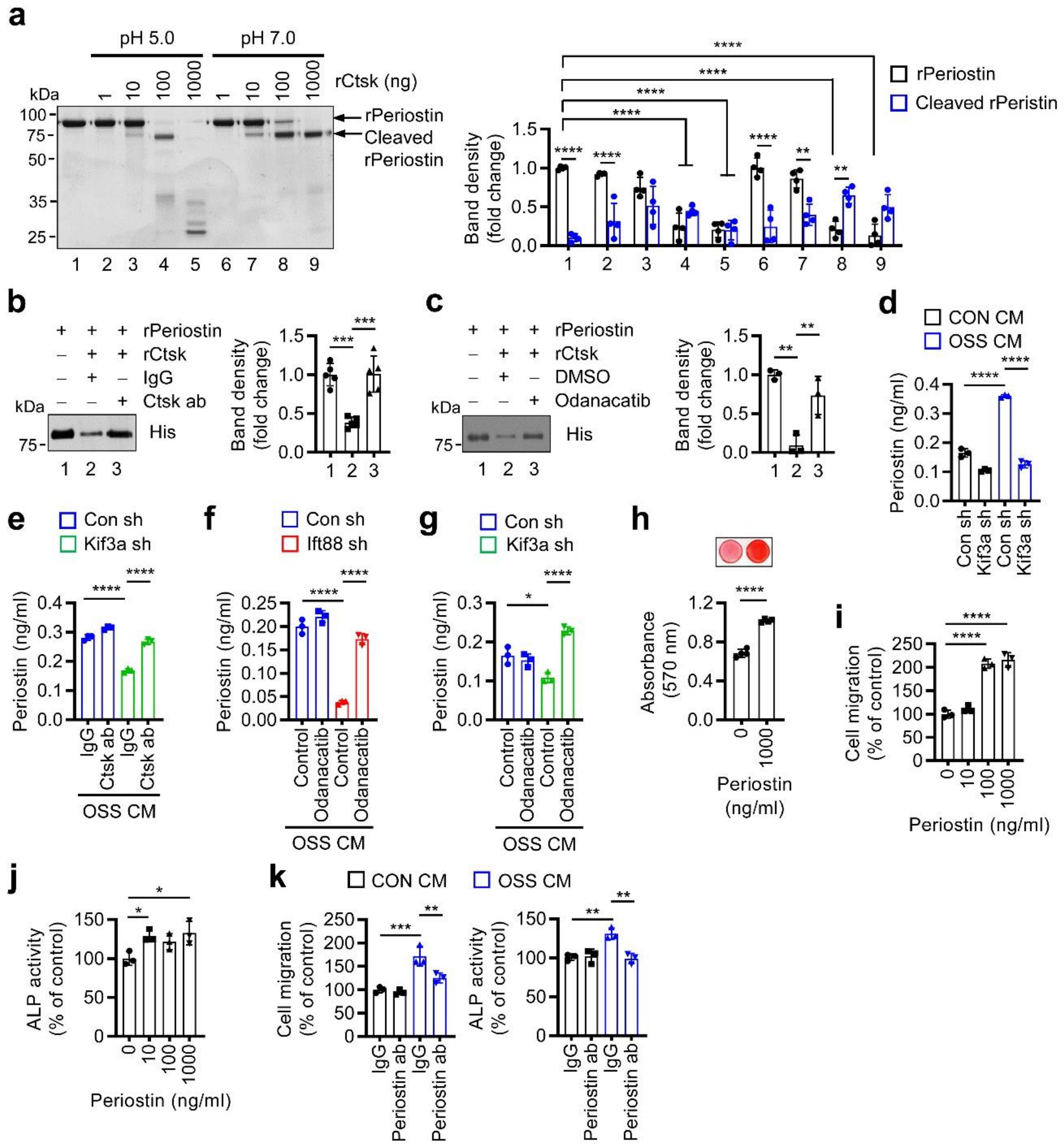

Supplementary Fig. 7. Shear stress-suppressed cathepsin K in preosteoclasts periostin-dependently increased migration and differentiation of osteoblasts. a His-tagged recombinant periostin (rPeriostin) was incubated with recombinant cathepsin K (rCtsk) at pH 5.0 and pH 7.0. Coomassie brilliant blue staining was performed, and their band densities are shown in the right (*n*=4). **b** and **c** His-rPeriostin was incubated with rCtsk and/or (**b**) 10 µg/mL Ctsk blocking antibody or (**c**) 100 nM Odanacatib in pH 7.0. Western blots were performed, and their band densities are shown in the right (*n*=5 **b**, *n*=3 **c**). **d** Preosteoclasts transfected with *Kif3a* sh were exposed to orbital shear stress (OSS) for 24 h or not (CON), and periostin levels were determined in their conditioned media (CM) by ELISA (*n*=3). **e–g** Preosteoclasts transfected with (**e** and **g**) *Kif3a* sh or (**f**) *Ift88* sh were exposed to OSS for 24 h in the presence or absence of (**e**) 10 µg/ml Ctsk antibody and (**f** and **g**) 100 nM Odanacatib. Periostin levels were determined in their CMs by ELISA (*n*=3, **e–g**). **h–j** Calvaria osteoblast precursors were treated with periostin at the indicated concentrations, and (**h**) their mineralization (*n*=4), (**i**) migration (*n*=3), and (**j**) alkaline phosphatase (ALP) activity (*n*=3) were measured. **k** Preosteoclasts were exposed to OSS for 24 h and then collected CM. Calvaria osteoblast precursors were treated with the CM in the presence of 5 µg/mL periostin blocking antibody. Their migration and ALP activity were measured (*n*=3). Mean±SD are

shown. Statistical significance was determined by Student's unpaired two-tailed t-test (**h**). One-way ANOVA was performed followed by Turkey's test (**b–g**, **i–k**). Two-way ANOVA was performed followed by Sidak's post-hoc test (**a**). $*P < 0.05$, $**P < 0.01$, $***P < 0.001$, and $****P < 0.0001$.

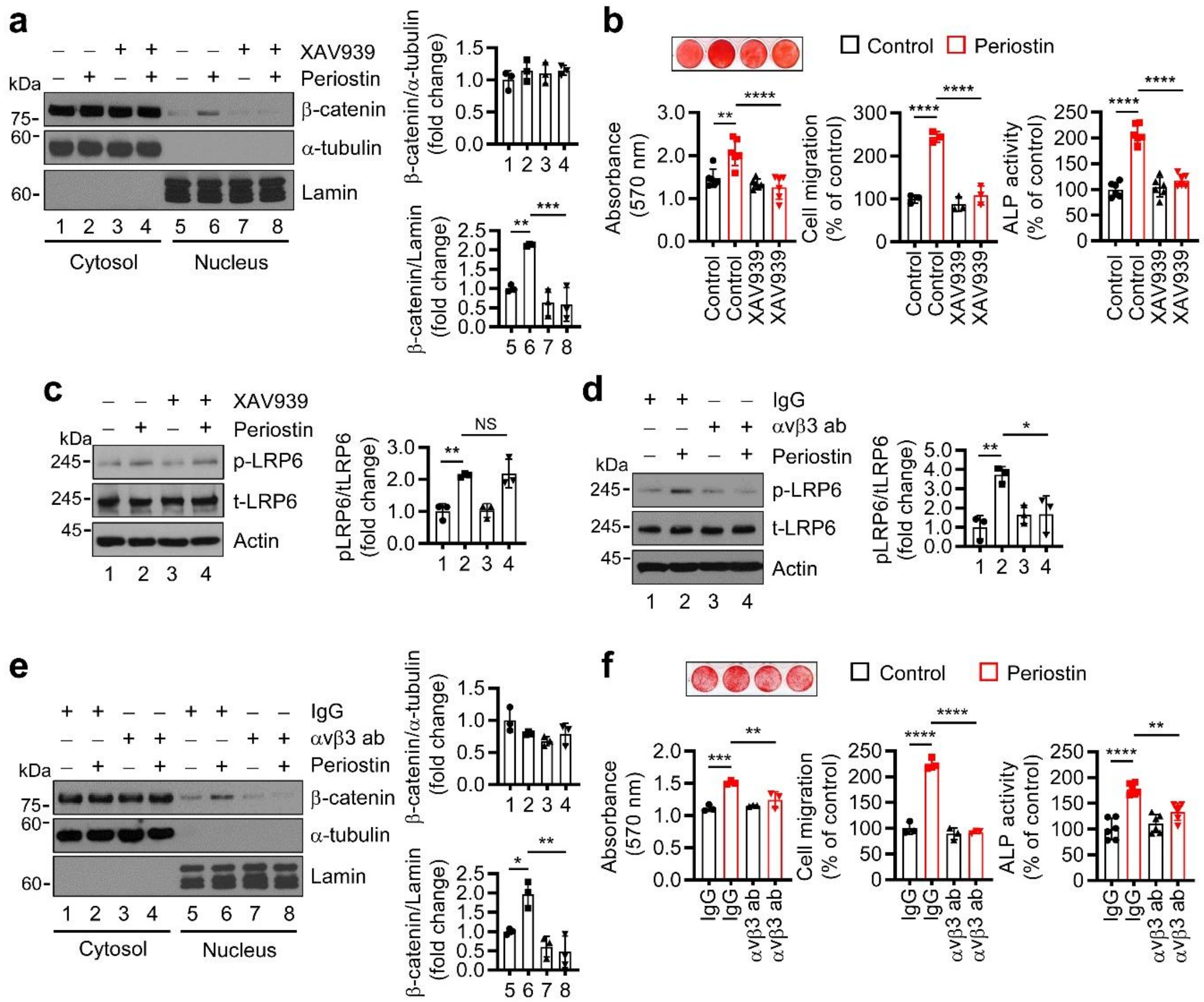

Supplementary Fig. 8. Periostin increased osteoblastic bone formation by activation of integrin αvβ3-LRP6-β-catenin signaling. a Calvaria osteoblast precursors were treated with 1 μg/mL recombinant periostin for 24 h after the pre-treatment with 1 μM XAV939. Their cytosolic and nuclear fractions were analyzed by western blot, and their band densities are shown in the right (*n*=3). **b** MC3T3-E1 cells (mineralization) or calvaria osteoblast precursors (migration and alkaline phosphatase (ALP) activity) were treated with 1 μg/mL recombinant periostin after the pre-treatment with 1 μM XAV939. Their mineralization (*n*=6), migration (*n*=3), and ALP activity (*n*=6) were measured. **c** and **d** Calvaria osteoblast precursors were treated with 1 μg/mL recombinant periostin for 15 min after the pre-treatment with (**c**) 1 μM XAV939 or (**d**) 10 μg/mL αvβ3 blocking antibody. Western blots were performed and their band densities are shown in the right (*n*=3). NS, not significant. **e** After pre-treatment with 10 μg/mL αvβ3 blocking antibody instead of XAV939, samples of osteoblast precursors were prepared as described in (**a**)**.** Band densities are shown in the right (*n*=3). **f** MC3T3-E1 cells (mineralization) or calvaria osteoblast precursors (migration and ALP activity) were treated with 1 μg/mL recombinant periostin after the pre-treatment with 10 μg/mL αvβ3 blocking antibody, and their mineralization (*n*=3), migration (*n*=3), and ALP activity (*n*=6) were measured. Mean±SD are shown. One-way ANOVA was performed followed by Turkey's test. **P* < 0.05, ***P* < 0.01, ****P* < 0.001, and *****P* < 0.0001.

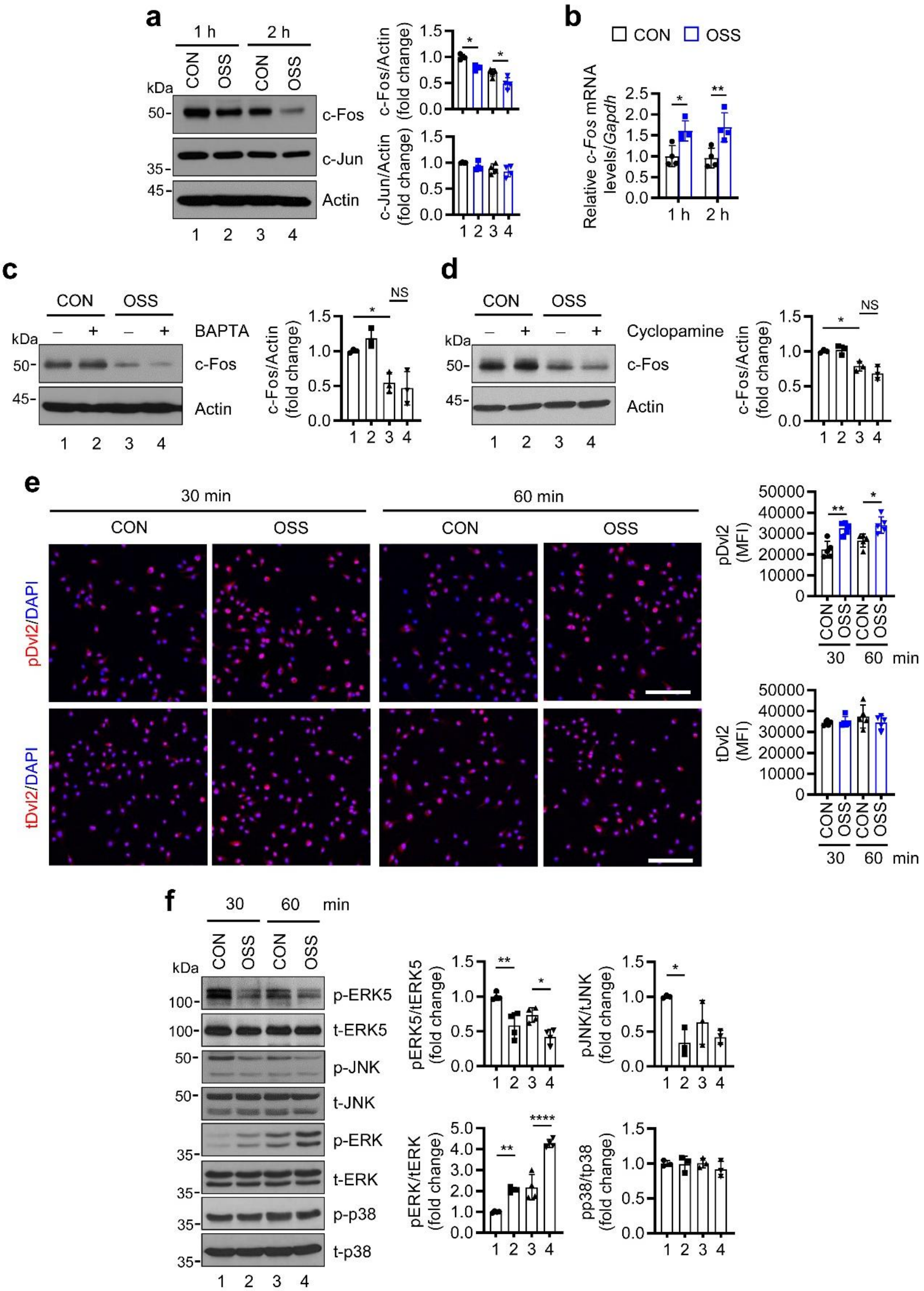

Supplementary Fig. 9. Primary cilia modulated expressions of c-Fos and phosphorylation of Dvl2, ERK5, and ERK in preosteoclasts in response to shear stress. a and **b** Preosteoclasts were exposed to orbital shear stress (OSS) for the indicated times or not (CON), and analyzed by (**a**) western blot and (**b**) real-time PCR (all,

n=4). **c** and **d** Preosteoclasts were treated with (**c**) 10 μM BAPTA and (**d**) 1 μM cyclopamine for 6 h, and then exposed to OSS for 2 h. The cells were analyzed by western blot (all, *n*=3). NS, not significant. **e** Immunostainings of phosphorylated Dvl2 (pDvl2) and total Dvl2 (tDvl2) in preosteoclasts exposed to OSS for the indicated times. Scale bar, 100 μm. Quantifications of the mean fluorescence intensity (MFI) of Dvl2 expression are shown in the right (*n*=5). **f** Preosteoclasts were exposed to OSS for the indicated times, and subjected to western blot (p-ERK5 and p-ERK: *n*=4; and p-JNK and p-p38: *n*=3). Band densities are shown in the right of each. Mean±SD are shown. One-way ANOVA was performed followed by Turkey's test (**a**, **c–f**). Two-way ANOVA was performed followed by Sidak's post-hoc test (**b**). *$P < 0.05$, **$P < 0.01$, and ****$P < 0.0001$.

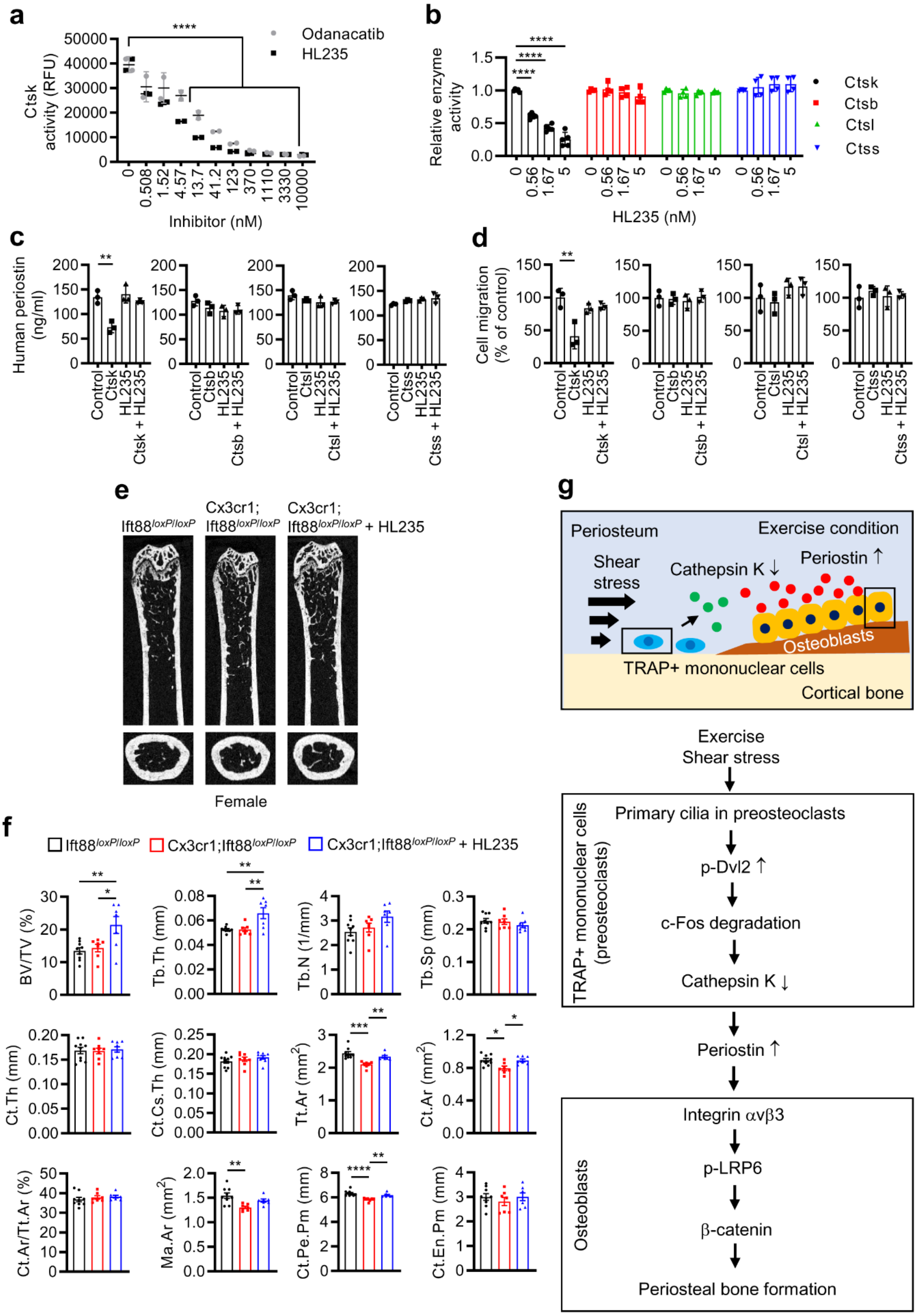
a
Ctsk activity (RFU)
Odanacatib
HL235
Inhibitor (nM)
b
Relative enzyme activity
Ctsk
Ctsb
Ctsl
Ctss
HL235 (nM)
c
Human periostin (ng/ml)
Control
Ctsk
HL235
Ctsk + HL235
Ctsb
Ctsb + HL235
Ctsl
Ctsl + HL235
Ctss
Ctss + HL235
d
Cell migration (% of control)
e
Ift88loxP/loxP
Cx3cr1; Ift88loxP/loxP
Cx3cr1; Ift88loxP/loxP + HL235
Female
f
Ift88loxP/loxP
Cx3cr1;Ift88loxP/loxP
Cx3cr1;Ift88loxP/loxP + HL235
BV/TV (%)
Tb.Th (mm)
Tb.N (1/mm)
Tb.Sp (mm)
Ct.Th (mm)
Ct.Cs.Th (mm)
Tt.Ar (mm2)
Ct.Ar (mm2)
Ct.Ar/Tt.Ar (%)
Ma.Ar (mm2)
Ct.Pe.Pm (mm)
Ct.En.Pm (mm)
g
Periosteum
Exercise condition
Shear stress
Cathepsin K ↓
Periostin ↑
Osteoblasts
TRAP+ mononuclear cells
Cortical bone
Exercise
Shear stress
TRAP+ mononuclear cells (preosteoclasts)
Primary cilia in preosteoclasts
p-Dvl2 ↑
c-Fos degradation
Cathepsin K ↓
Periostin ↑
Osteoblasts
Integrin αvβ3
p-LRP6
β-catenin
Periosteal bone formation

Supplementary Fig. 10. Ctsk inhibition recovered cortical bone size in exercising female Cx3cr1;Ift88$^{loxP/loxP}$ mice. **a** Cathepsin K (Ctsk) activity was measured in the presence of Odanacatib and HL235 at the indicated concentrations (*n*=2). **b** Enzymatic activity assays of Ctsk (*n*=5), cathepsin B (Ctsb, *n*=4), cathepsin L (Ctsl, *n*=4), and cathepsin S (Ctss, *n*=4) measured using isoform-specific fluorometric substrates in the presence of HL235. **c** and **d** Quantification of periostin levels remaining in the lower chamber after the migration assay using a human periostin ELISA kit (*n*=3) (**c**). Migration assay of periosteum-derived progenitor cells toward CMs from preosteoclasts pretreated with human periostin (0.6 μg) in the presence or absence of Ctsk, Ctsb, Ctsl, or Ctss (10 ng each), with or without HL235 (1 μM) (*n*=3) (**d**). **e** and **f** After administering tamoxifen, female Cx3cr1;Ift88$^{loxP/loxP}$ mice and their Ift88$^{loxP/loxP}$ littermates at 12 weeks of age were exercised on a treadmill for 4 weeks as described in the Methods. The Cx3cr1;Ift88$^{loxP/loxP}$ mice were orally injected or not with 100 μL HL235 (100 mg/kg) twice a week. (**e**) Representative micro-CT images of the proximal femurs of the Ift88$^{loxP/loxP}$ (*n*=9), Cx3cr1;Ift88$^{loxP/loxP}$ (*n*=7), and HL235 treated Cx3cr1;Ift88$^{loxP/loxP}$ (*n*=7) mice at 16 weeks of age are shown. (**f**) Their quantitative results are shown. BV/TV, trabecular bone volume per tissue volume; Tb.Th, trabecular thickness; Tb.N, trabecular number, Tb.Sp, trabecular spacing; Ct.Th, cortical thickness; Ct.Cs.Th, cortical cross-sectional thickness; Tt.Ar, total cross-sectional area inside the periosteal envelope; Ct.Ar, cortical area; Ma.Ar, marrow area; Ct.Pe.Pm, cortical periosteal perimeter; and Ct.En.Pm, cortical endosteal perimeter. **g** A proposed model for primary cilia of preosteoclasts that stimulate periosteal bone formation. Activated primary cilia by mechanical stimuli, such as exercise and shear stress, reduce production and secretion of Ctsk production. Increased extracellular periostin due to decreased degradation of extracellular periostin by Ctsk increases bone formation in osteoblasts at periosteal surface. Mean±SD (**a**–**d**) or mean±SEM (**f**) are shown. One-way ANOVA was performed followed by Turkey's test (**c**,**d**,**f**). Two-way ANOVA was performed followed by Sidak's post-hoc test (**a**,**b**). *$P < 0.05$, **$P < 0.01$, ***$P < 0.001$, and ****$P < 0.0001$
